# The evolution of chemical ordering and property in Fe_1+*x*_Se_2_ upon intercalation ratios

**DOI:** 10.1093/nsr/nwae430

**Published:** 2024-11-29

**Authors:** Zijing Zhao, Xiaocang Han, Shengcai Zhu, Zhi Fang, Ziyi Han, Zhongyu Liang, Bailing Li, Biao Zhang, Wei Li, Zhaochu Luo, Licong Peng, Xiaoxu Zhao, Xiangguo Li, Jiadong Zhou, Song Gao, Chengxin Wang, Mathias Kläui, Yanglong Hou

**Affiliations:** School of Materials Science and Engineering, Beijing Key Laboratory for Magnetoelectric Materials and Devices, Peking University, Beijing 100871, China; School of Materials, Shenzhen Campus of Sun Yat-sen University, Shenzhen 518107, China; School of Physics and Optoelectronic Engineering, Beijing University of Technology, Beijing 100124, China; School of Materials Science and Engineering, Beijing Key Laboratory for Magnetoelectric Materials and Devices, Peking University, Beijing 100871, China; School of Materials, Shenzhen Campus of Sun Yat-sen University, Shenzhen 518107, China; School of Materials Science and Engineering, Beijing Key Laboratory for Magnetoelectric Materials and Devices, Peking University, Beijing 100871, China; School of Materials Science and Engineering, Beijing Key Laboratory for Magnetoelectric Materials and Devices, Peking University, Beijing 100871, China; State Key Laboratory for Artificial Microstructure & Mesoscopic Physics, School of Physics, Peking University, Beijing 100871, China; School of Materials Science and Engineering, Beijing Key Laboratory for Magnetoelectric Materials and Devices, Peking University, Beijing 100871, China; School of Materials Science and Engineering, Beijing Key Laboratory for Magnetoelectric Materials and Devices, Peking University, Beijing 100871, China; School of Materials Science and Engineering, Beijing Key Laboratory for Magnetoelectric Materials and Devices, Peking University, Beijing 100871, China; State Key Laboratory for Artificial Microstructure & Mesoscopic Physics, School of Physics, Peking University, Beijing 100871, China; School of Materials Science and Engineering, Beijing Key Laboratory for Magnetoelectric Materials and Devices, Peking University, Beijing 100871, China; School of Materials Science and Engineering, Beijing Key Laboratory for Magnetoelectric Materials and Devices, Peking University, Beijing 100871, China; School of Materials, Shenzhen Campus of Sun Yat-sen University, Shenzhen 518107, China; Key Lab of Advanced Optoelectronic Quantum Architecture and Measurement (MOE), School of Physics, Beijing Institute of Technology, Beijing 100081, China; School of Chemistry, Sun Yat-sen University, Guangzhou 510275, China; School of Materials Science and Engineering, Sun Yat-sen University, Guangzhou 510275, China; Institute of Physics, Johannes Gutenberg University Mainz, Mainz 55128, Germany; School of Materials Science and Engineering, Beijing Key Laboratory for Magnetoelectric Materials and Devices, Peking University, Beijing 100871, China; School of Materials, Shenzhen Campus of Sun Yat-sen University, Shenzhen 518107, China

**Keywords:** intercalation, Fe-intercalated 2D materials, room-temperature magnetism

## Abstract

Intercalation has been considered as an effective method to explore innovative two-dimensional (2D) materials and modify their properties. However, the relationship between intercalation concentration, structure, and property remains a largely uncharted territory, and the controllable synthesis of desired intercalated phases faces challenges. Here, a general intercalated rule for the effect of self-intercalation ratio on atomic arrangements is revealed. Then, the controllable synthesis of a series of Fe-intercalated 2D materials is realized. Scanning transmission electron microscopy illustrates that their intercalation structures undergo disordered/ordered/half-ordered/ordered transformation, which confirms the intercalated rule and proposes a new structure termed half-ordered intercalation. Notably, their magnetic and electrical properties can be significantly modulated by intercalation. Orderly intercalated nanoflakes possess room-temperature magnetism with composition-regulated magnetic domains. Moreover, Fe_1.5_Se_2_ and Fe_1.6_Se_2_ are scarce half-metallic materials showing different magneto-resistance behaviors. This work would guide the design and synthesis of new intercalated materials, and deepen the understanding of the relationship between structure and properties.

## Teaser

With increasing the intercalation ratios, the intercalated structures of Fe(1+x)Se2 undergo the disordered/ordered/half-ordered/ordered transition, accompanied by the regulation of the magnetic and electrical properties.

## INTRODUCTION

Two-dimensional (2D) materials have provoked a surge of interest as they offer unprecedented opportunities for exploring unique phenomena and designing advanced low-consumption, highly integrated, and flexible devices [1–3]. Nevertheless, the category of discovered 2D materials (especially for 2D room-temperature magnetic materials) is still limited, which greatly hinders their practical applications [4]. Additionally, the structure–activity relationship between atomic arrangements and properties, which is helpful in guiding material design and property modification, remains unclear.

Recently, intercalating native metal atoms (self-intercalation) into the van der Waals (vdW) gaps of transition metal dichalcogenides (TMD) has been considered to be an effective method to create novel 2D materials [5–9]. It can also induce newfangled phenomena while maintaining the outstanding characteristics of its 2D parent structure [10–12]. The properties can be regulated by changing the intercalation ratio (IR) or ordering. For instance, 16.7% Ta intercalation into vdW gaps of nonmagnetic TaS_2_ can induce the appearance of magnetism [5]. Cr_3_Te_4_, which can be viewed as 50% Cr self-intercalation into T phase CrTe_2_, showed biskyrmionic bubbles owing to the magnetic interactions between pristine Cr atoms in the CrTe_2_ backbone and the intercalated Cr atoms [13]. Trigonal Cr_5_Te_8_ with a self-intercalation ratio of 25% showed strong perpendicular magnetic anisotropy, while monoclinic Cr_5_Te_8_ in different intercalated structures exhibited colossal anomalous Hall conductivity and Hall angle [14]. However, the general relationship between self-intercalated concentration and atomic structure remains largely unexplored, but it is of significant importance for designing new materials and predicting their structures. Moreover, it is also extremely desirable to reveal the influence of intercalated structures on the physicochemical properties in order to further improve their performance. Last but not least, previous studies mostly focused on V/Nb/Ta-based and Cr-based self-intercalated TMD [5,12,15–18], whose Curie temperatures are still below room temperature and magnetic properties are far from satisfactory for extending the applications [19].

Fe-based intercalated materials have come into our view due to their broad applications in high-temperature spintronics and data storage [20–23]. VdW FeSe_2_ is nonmagnetic with the phase transition at ∼11 K [24]. Nevertheless, the self-intercalation of Fe atoms (typical magnetic atoms with five unpaired electrons) into the FeSe_2_ interlayer may introduce spin polarization, offering a promising avenue to develop new categories of 2D magnets and delicately regulating spin structures. Surprisingly, 50% Fe-intercalated FeSe_2_ (i.e. Fe_3_Se_4_) shows a huge coercivity and high energy product [25,26], which is a potential low-cost compound to replace rare earth or noble metal magnets. More importantly, it is predicted that Fe_3_Se_4_ is a scarce magnetic half-metal, showing immense promise in magnetic tunnel junctions [27]. However, the exploration of its electrical properties has yet to be unveiled. Despite the exceptional properties exhibited by Fe-based self-intercalated materials, current research about them remains limited. Besides, Fe-Se compounds have a lot of stoichiometric proportions and structures, so controllably synthesizing the desired intercalation phase to obtain fascinating properties also faces huge challenges.

In this work, we reveal the general rule of the relationship between IR and intercalated ordering in T phase self-intercalated TMD, and predict various possible intercalation structures. Through precisely regulating the metal chemical potential in the confined reaction space, a series of Fe self-intercalated 2D nanoflakes with an IR of 18% (Fe_1.18_Se_2_, new materials), 25% (Fe_1.25_Se_2_), 50% (Fe_1.5_Se_2_), 60% (Fe_1.6_Se_2_, new materials), and 75% (Fe_1.75_Se_2_), are controllably synthesized. Scanning transmission electron microscopy (STEM) images show that their structures undergo the disordered/ordered/half-ordered/ordered transition, which is consistent with the predicted intercalation rule. Notably, the magnetic and electrical properties of Fe_1+*x*_Se_2_ are significantly affected by IR. Fe intercalation induces charge transfer and spin polarization into nonmagnetic FeSe_2_, and all orderly intercalated 2D nanoflakes exhibit room-temperature magnetism with the thickness even down to ∼5 nm. Additionally, Fe_1.5_Se_2_ and Fe_1.6_Se_2_ are demonstrated to be rare magnetic half-metals with different spin gaps. Fe_1.5_Se_2_ exhibits the transition from negative magneto-resistance (MR) to positive MR with decreasing temperatures, while Fe_1.6_Se_2_ maintains positive MR. This work enriches the intercalated system and the 2D magnetic family, and provides a classic paradigm for structural modulation of magnetic and electric properties.

## RESULTS AND DISCUSSION

### The general intercalation rule between intercalation ratio and atomic ordering

**Figure 1.**
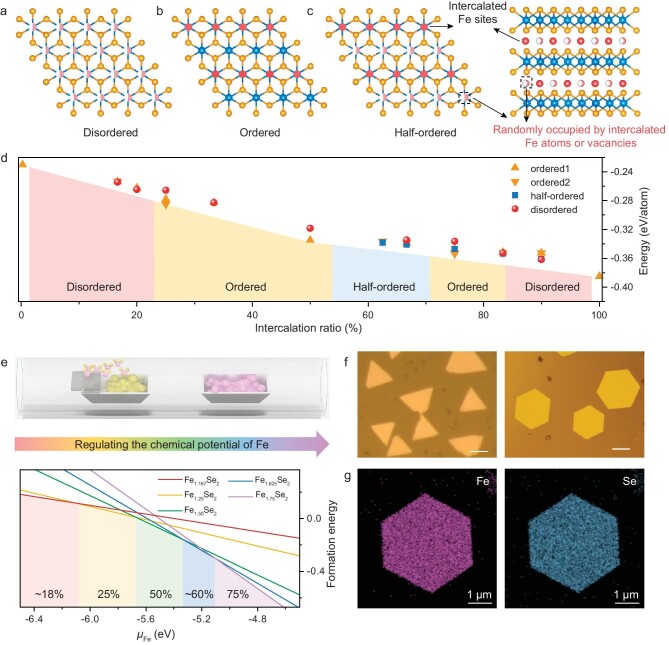
Controllable synthesis of 2D Fe_1+*x*_Se_2_ nanoflakes. (a–c) Atomic models of disordered intercalation (a), ordered intercalation (IR = 50%) (b), and half-ordered intercalation (c) structures. Blue balls indicate the Fe atoms in the FeSe_2_ backbone, and yellow balls represent Se atoms. Red balls indicate the intercalated Fe sites with full occupation, while pink and white balls indicate that the sites are randomly occupied by intercalated Fe atoms or vacancies with disordered arrangement. (d) The formation energy of Fe_1+*x*_Se_2_ with various intercalation ratios in different intercalated structures. (e) Schematic for the composition-controlled growth of 2D Fe_1+*x*_Se_2_ nanoflakes by regulating chemical potential; the formation energy is correlated with the chemical potential of Fe (${{\mu }_{Fe}}$). (f) Optical microscopy (OM) images of Fe_1+*x*_Se_2_ nanoflakes with triangular or hexagonal shapes. Scale bars: 10 μm. (g) EDS mapping images of Fe and Se in Fe_1+*x*_Se_2_, respectively.

As mentioned above, the concentrations and arrangements of intercalation atoms within the van der Walls (vdW) gaps of TMD will have a significant impact on their properties. Here, we initially focus on the effect of IRs on the atomic structures in T phase TMD. Fe_1+*x*_Se_2_ is selected as the representative self-intercalation system, where native Fe atoms are intercalated in the T phase FeSe_2_ interlayers when regarding FeSe_2_ as the backbone structure. Then, we calculate the energies of ordered, disordered, and half-ordered intercalation structures with various IRs. In the disordered structures, intercalated atoms are randomly distributed (Fig. 1a). In contrast, ordered intercalation structures exhibit well-defined superlattice topological patterns (Fig. S1 and Fig. 1b). Specifically, as for 1/*N* (*N* = 2, 3, 4, 6) ordered supercells (Fig. S1a–e), intercalated sites are separated by *N* − 1 atoms along the direction of the nearest Fe atoms (the red arrow in Fig. S1a). These intercalation sites can also be rearranged to form other highly symmetrical and ordered supercells (Fig. S1f, g). For (*N* − 1)/*N* ordered intercalation, non-intercalated sites are arranged at the equivalent intercalated sites in the 1/*N* structure (Fig. S1h–k). Notably, a new structure termed half-ordered intercalation is proposed, where the intercalated atoms in half of the intercalation sites are ordered, while atoms in the remaining half are randomly distributed (Fig. 1c).

As shown in Fig. 1d, when the IR in Fe_1+*x*_Se_2_ is lower than ∼0.2, the disordered intercalation structure is the most stable. This can be attributed to the fact that the distance between the nearest intercalated Fe atoms is too long to influence each other, resulting in a disordered state. As the intercalation concentration increases (IR reaches ∼0.25), the intercalated Fe atoms prefer forming an ordered superlattice. When ∼0.5 < IR < ∼0.68, it tends to form half-ordered structures (i.e. half of the intercalation sites are fully occupied while the remaining half are occupied randomly). If ∼0.68 < IR < ∼0.87, intercalation sites become ordered to form a superlattice. Interestingly, a similar intercalation rule is also observed in Ti_1+_*_x_*S_2_ and V_1+_*_x_*Te_2_ systems (Fig. S2). This indicates that such a rule could be generalized when the backbone has a similar topological structure. Therefore, the intercalation rule can be summarized: (1) Intercalation atoms prefer to form supercells with higher symmetry along the nearest metal sites due to the largest interaction, and Fig. S1 gives almost all ordered arrays of intercalation states in T phase TMD. (2) The thermodynamically stable intercalation structure depends on the concentration of self-intercalated atoms—it changes from disordered, to ordered, then to half-ordered (IR >0.5), back to ordered, and further to disordered structure in T phase self-intercalated TMD, with an increase in the IR. The critical ratio varies with elemental composition. (3) If the energy difference between different intercalation states is small, the entropy change needs to be taken into account, so the specific synthesis process (growth temperature, annealing, *etc.*) will affect the final intercalated structure as well.

### The controllable synthesis of intercalated Fe_1+*x*_Se_2_ nanoflakes

In order to confirm the above intercalation rule, we synthesized a family of Fe_1+*x*_Se_2_ with different IRs and explored their intercalated structures. The Gibbs free energy of formation (Δ*G*) of Fe_1+*x*_Se_2_ was investigated to explore the growth window. According to the theory of chemical thermodynamics, when temperature (*T*) and pressure (*P*) are constant during the growth process, Δ*G* can be expressed as ${\mathrm{\Delta }}G = G( {{\mathrm{F}}{{{\mathrm{e}}}_{1 + x}}{\mathrm{S}}{{{\mathrm{e}}}_2}} ) - G( {{\mathrm{FeS}}{{{\mathrm{e}}}_2}} ) - x{{\mu }_{{\mathrm{Fe}}}}$, where ${{\mu }_{{\mathrm{Fe}}}}$ represents the chemical potential of Fe atoms. The formation energy versus chemical potential phase diagram is constructed based on density functional theory (DFT) calculations (Fig. 1e), showing that the most stable material (with the lowest energy) changes from Fe_1.167_Se_2_ (∼18% intercalation), to Fe_1.25_Se_2_ (25% intercalation), then to Fe_1.5_Se_2_ (50% intercalation), subsequently to Fe_1.625_Se_2_ (∼60% intercalation), and further to Fe_1.75_Se_2_ (75% intercalation), with increasing chemical potential of Fe. Therefore, even minor variations in the growth conditions that affect the chemical potential can significantly affect the IR and composition-controlled synthesis.

Given this, the space confinement-assisted chemical potential regulation strategy was utilized for the synthesis of Fe_1+*x*_Se_2_ nanoflakes. Two mica substrates are stacked face-to-face to create a micro confined reaction space [28], which is used to provide a relatively stable and uniform gas flow to precisely control the chemical potential during growth, facilitating the formation of ultrathin pure-phase crystals (Fig. S3). By decreasing the volatilization temperature or mass of the Se source and keeping other growth conditions unchanged in the chemical vapor deposition (CVD) growth process, the chemical potential of the precursor is regulated and the IR is increased (Fig. S4). Specifically, the concentration of Se in the CVD tube decreases during this process. Simultaneously, the volatility of FeCl_2_ precursor is improved (surface is less poisoned by Se), so the concentration of Fe vapor is increased. Thus, the molar fraction of Fe (i.e. the ratio of the moles of Fe to the total moles of all components) in the CVD tube is greatly increased. Additionally, chemical potential is expressed as $\mu ( {T,P} ) = {{\mu }^*}( {T,P} ) + RT\mathrm{ln}y$, where ${{\mu }^*}( {T,P} )$ is a constant at a certain pressure and temperature, *R* is the molar gas constant, and *y* represents the molar fraction. As a result, the chemical potential of metal is augmented, facilitating an increased degree of self-intercalation and phase evolution, as calculated in (Fig. 1e). Besides, the nanoflakes exhibit a transition from triangular to hexagonal shapes (Fig. 1f). Energy dispersive spectroscopy (EDS) (Fig. 1g and Fig. S5) only detects the signals of Fe and Se elements in these Fe_1+*x*_Se_2_ nanoflakes with different atomic ratios.

### The intercalation structures of Fe_1+*x*_Se_2_

**Figure 2.**
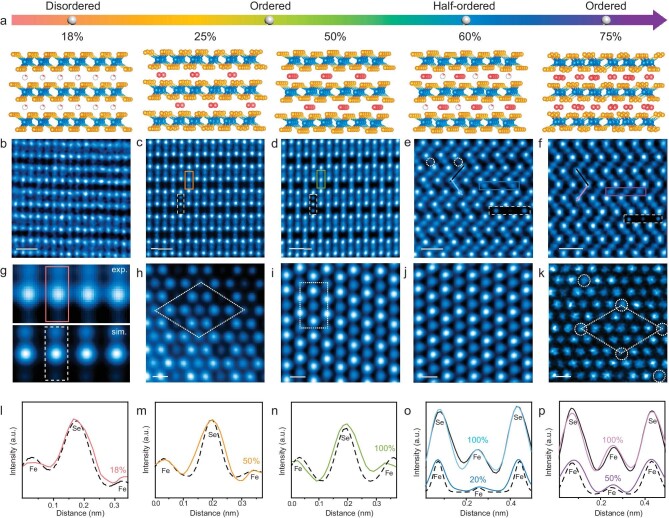
Atomic structures of 2D Fe_1+*x*_Se_2_ nanoflakes. (a) Atomic models of Fe_1+*x*_Se_2_ with various intercalation ratios. Blue and yellow balls represent the Fe and Se atoms in the FeSe_2_ backbone, respectively. The ordered (disordered) intercalated Fe atoms are denoted by red (red and white) balls. (b–f) Cross-sectional atomic-resolution ADF-STEM images of 2D Fe_1+*x*_Se_2_ (*x* = 0.18, 0.25, 0.5, 0.6, 0.75) nanoflakes. White circles indicate the additional intercalation located at Fe_B_ sites. Scale bars: 0.5 nm. (g) Averaged STEM images of pristine Fe-Se-intercalated Fe sites from experimental (exp.) and simulated (sim.) Fe_1.18_Se_2_. (h–k) Top-view atomic-resolution STEM images of 2D Fe_1+*x*_Se_2_ (*x* = 0.25, 0.5, 0.6, 0.75) nanoflakes. Dashed lines indicate rhombic or rectangular symmetry. Scale bars: 0.2 nm. (l–p) Intensity line profiles from the experimental (marked by solid lines) and corresponding simulated images (marked by dashed lines) inserted in (g, c–f). Colored, black, and dashed black lines represent profiles from experimental intercalation layers, pristine Se-Fe-Se layer (black lines), and simulated images (dashed lines), respectively.

Aberration-corrected annular dark-field scanning transmission electron microscopy (ADF-STEM) was employed to unveil the atomic structures and intercalation ratios of self-intercalated Fe_1+*x*_Se_2_ crystals (Fig. 2a). Representative atomic-resolution ADF-STEM images of 2D Fe_1+*x*_Se_2_ nanoflakes are taken along the *a*-axis (Fig. 2b–d) or *b*-axis (Fig. 2e, f). These cross-sectional STEM images consistently show intercalated Fe atoms fill the vdW gaps of FeSe_2_, where backboned Fe atoms (darker blobs) are sandwiched between top and bottom Se atoms (brighter contrast). The intensity variations of intercalated sites within Fe_1+*x*_Se_2_ indicate different IRs, which could be quantitatively determined by comparing the peak intensities of the original backbone Fe (Fe_o_) and intercalated Fe (Fe_i_) in STEM images.

The low Fe-chemical potential produces a minimal extent of intercalation in FeSe_2_, as indicated by the faint contrast in the vdW gaps. As shown in Fig. 2b and Fig. S6, the intercalated Fe atoms display a disordered distribution. To quantify the intercalation concentration of Fe atoms, we implement a homemade program, which is capable of automatically identifying the intensity of the original backbone metal dots and intercalated metal columns [29]. Each bright spot is modeled as a superposition of 2D Gaussian functions, followed by averaging. We integrate and normalize the intensity of all intercalated Fe atomic sites in the experimental images, which is determined to be ∼18%, as corroborated by the consistency of the intensity line profiles between the experimental and simulated images (Fig. 2g, l).

When increasing the chemical potential of Fe during the growth process, ordered intercalation can be achieved (Fig. 2c–f), exhibiting different periodicity as further illustrated in top-view STEM images (Fig. 2h–k). The consistency of the intensity at intercalated sites between experimental and simulated images (or backboned FeSe_2_) demonstrates the Fe intercalated ratios (Fig. 2m–p). The intercalated Fe atoms in Fe_1.25_Se_2_ (Fe_1.5_Se_2_) occupy the octahedral vacancies, filling every second vacancy (Fig. 2c, d). As shown in Fig. 2m and n, the intensity line profiles of experimental intercalated Fe (colored lines) are consistent with the intensity of simulated 50% or 100% Fe intercalation (dashed lines), demonstrating the total intercalated ratios of 25% and 50%, respectively. The phase is also distinguished by the periodic arrangements of intercalated atoms exhibiting rhombic or rectangular geometries from top-view STEM images (Fig. 2h, i, and Figs S7, S8).

When the IR is further increased (Fig. 2e, j), intercalated atoms present similar arrays to Fe_1.5_Se_2_ (Fig. S9) with 100% intercalation at Fe_A_ sites. The difference lies in an additional ∼20% average intercalation at Fe_B_ sites, which exhibit a relatively random arrangement (Fig. 2o). It is worth noting that the intercalated Fe atoms at Fe_B_ sites are inhomogeneous and disordered at the atomic scale (Fig. S10). In this regard, Fe_1.6_Se_2_ nanoflakes exhibit an average IR of ∼60% with a half-ordered structure, representing a new intercalation configuration. Moreover, the intercalated atomic lattice in Fe_1.75_Se_2_ is shown in Fig. 2f, k, with 100% intercalation at one kind of site and ordered 50% intercalation at another kind of site, due to the coherence observed in the intensity line profiles between experimental intercalated Fe (colored lines) and backbone 100% Fe (black line) or simulated 50% Fe (dashed line) in Fig. 2p.

To further analyze the intercalated structures, the corresponding selected area electron diffraction (SAED) patterns of Fe_1+*x*_Se_2_ are shown in Fig. S11. The diffraction spots of 18% intercalated samples (Fe_1.18_Se_2_) all originate from the FeSe_2_ backbone, indicating the disordered arrangement of intercalated atoms as well. Nevertheless, 25% and 75% intercalated nanoflakes (Fe_1.25_Se_2_, Fe_1.75_Se_2_) show additional superspots (denoted by white and purple circles), signifying the orderly intercalation of Fe atoms between FeSe_2_ vdW gaps [22,30]. The diffraction patterns of Fe_1.5_Se_2_ and Fe_1.6_Se_2_ exhibit similar characteristics, implying that they have the same periodic structure and the intercalation atoms at Fe_B_ sites of Fe_1.6_Se_2_ are disordered. Therefore, by employing atomic-scale STEM and SAED analysis, we have proved that the as-synthesized 2D Fe_1+*x*_Se_2_ crystals go across disordered (∼18%), ordered (25%, 50%), half-ordered (∼60%), and then to ordered (75%) arrays, by increasing the intercalation concentration.

Besides, these nanoflakes exhibit different Raman vibration peaks (Fig. S12), which provides an efficient and nondestructive way to distinguish the phases quickly. X-ray diffraction (XRD) characterization is also performed to explore the intercalated structures (Fig. S13). As the intercalation concentration increases, the peaks shift towards lower XRD angles, indicating that the interlayer gaps increase gradually and the samples are in pure phases. The thickness of Fe_1+*x*_Se_2_ can be regulated by adjusting the growth time (Fig. S14).

### Intercalation-regulated magnetic and electrical properties

**Figure 3.**
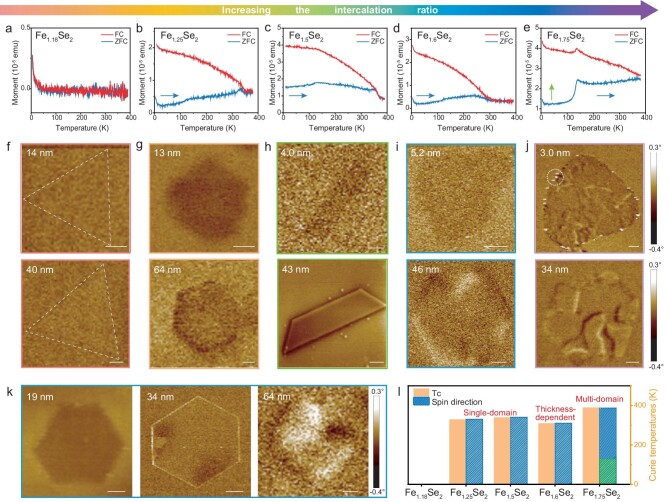
Magnetic properties of Fe_1+*x*_Se_2_ nanoflakes. (a–e) Magnetization versus temperature curves of Fe_1.18_Se_2_, Fe_1.25_Se_2_, Fe_1.5_Se_2_, Fe_1.6_Se_2_, and Fe_1.75_Se_2_ nanoflakes with the in-plane magnetic field of 500 Oe, respectively. The arrows indicate that the spins are along the *c*-plane or *c*-axis directions. (f) MFM phase images of Fe_1.18_Se_2_ nanoflakes with different thicknesses, showing no magnetic domains. (g) MFM phase images of Fe_1.25_Se_2_ nanoflakes, showing the single-domain states. (h) MFM phase images of Fe_1.5_Se_2_ nanoflakes with single-domain states. (i) MFM phase images of Fe_1.6_Se_2_ nanoflakes, showing different magnetic domain states varying with thickness. (j) MFM phase images of Fe_1.75_Se_2_ nanoflakes, exhibiting the multiple domain states. Scale bars: 1 μm. (k) Fe_1.6_Se_2_ nanoflakes evolve from single to multiple domain structures with increase of thickness. Scale bars: 2 μm. (l) Comparison of Curie temperatures (*T*_C_), spin directions, and magnetic domains in Fe_1.18_Se_2_, Fe_1.25_Se_2_, Fe_1.5_Se_2_, Fe_1.6_Se_2_, and Fe_1.75_Se_2_. Blue columns indicate easy-axis is in the *c*-plane, and green columns indicate easy-axis is in the *c*-axis.

The magnetic properties of Fe_1+*x*_Se_2_ nanoflakes can be regulated by changing the intercalation structures. As shown in Fig. 3a–e and Fig. S15, Fe_1.18_Se_2_ is paramagnetic, while the samples with higher intercalation concentrations (>25%) show room-temperature (RT) magnetism. Therefore, a small number of intercalated atoms are not sufficient to introduce long-range magnetic coupling, but well-ordered intercalated magnetic atoms can induce spin polarization into the system. Moreover, the Curie temperature and spin directions also vary with the intercalated structure. In general, the Curie temperature of Fe_1+*x*_Se_2_ increases gradually by enhancing the IR, but Fe_1.6_Se_2_ exhibits a slight decrease, which may arise from the disordered array of intercalated Fe atoms at Fe_B_ sites in Fe_1.6_Se_2_ [31]. The spin directions of intercalated samples are in the *c*-plane as discussed below. It is worth noting that the zero-field cooling (ZFC) curve drops drastically and the field cooling (FC) curve exhibits a peak at ∼130 K for Fe_1.75_Se_2_ (Fig. 3e). This phenomenon indicates the unique spin-reorientation behavior transitioning from the *c*-plane to *c*-axis [32].

In addition, RT magnetic force microscopy (MFM) was used to elaborate on the magnetic domain structures of single Fe_1+*x*_Se_2_ nanosheets. Fig. 3f–j and Figs S16, S17 exhibit MFM images of Fe_1+*x*_Se_2_ nanoflakes with different thicknesses. Fe_1.18_Se_2_ shows no obvious magnetic phase signal (Fig. 3f), consistent with its paramagnetism. However, the highly intercalated samples have a pronounced darker contrast compared with the mica substrate (Fig. 3g–j). If the phase signal comes from the height response of surface topography, it will display a brighter contrast like the white circles marked in Fig. 3j. Therefore, the dark contrast of Fig. 3g–j results from the tip-sample magnetic interactions, further verifying the RT magnetic behavior of orderly intercalated Fe_1+*x*_Se_2_ nanoflakes. The weak phase contrast indicates the in-plane magnetism of samples as well. Notably, Fe_1.25_Se_2_ and Fe_1.5_Se_2_ exhibit single-domain magnetic structures in all thicknesses ranging from ∼5 nm to ∼190 nm (Fig. 3g, h and Fig. S17a). Nevertheless, Fe_1.75_Se_2_ shows multi-domain magnetic patterns with thickness even down to ∼3 nm (Fig. 3j). Interestingly, as for Fe_1.6_Se_2_, thinner nanoflakes are single domains, while thicker nanoflakes show multi-domain states characterized by alternating light and dark contrast of MFM phase images (Fig. 3i, k and Fig. S18). This thickness-dependent magnetic domain structure originates from the decrease of magnetostatic energy through reducing the thickness which cannot compensate for the increase of domain wall energy [33–35]. In other words, the RT magnetic domain structures of Fe_1+*x*_Se_2_ nanosheets evolve from single-domain to multi-domain states with the increase in intercalation concentration. In all, the magnetic properties of Fe_1+*x*_Se_2_ nanoflakes, including Curie temperatures, spin directions, and magnetic domain states, vary with the intercalated structures (Fig. 3l).

**Figure 4.**
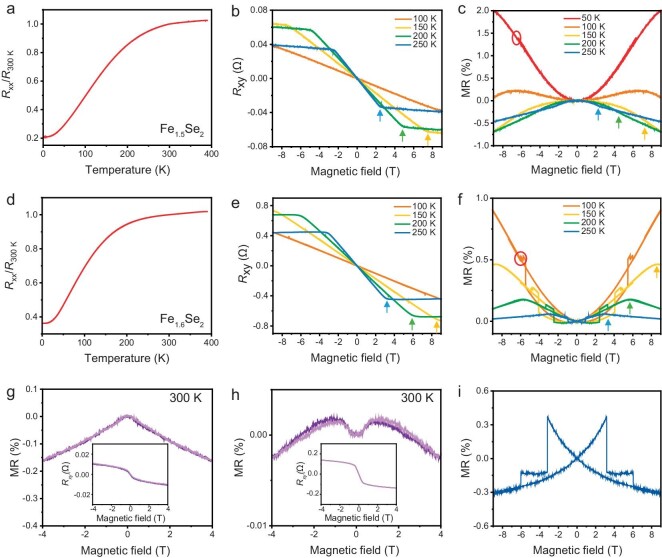
Electrical properties of Fe_1.5_Se_2_ and Fe_1.6_Se_2_ nanoflakes. (a) Typical temperature-dependent longitudinal resistance (*R*_xx_)/*R*_300 K_ of Fe_1.5_Se_2_ nanoflake. (b) Magnetic field-dependent Hall resistance (*R*_xy_) of Fe_1.5_Se_2_ at different temperatures. The arrows indicate the saturation magnetic fields of Fe_1.5_Se_2_. (c) Field-dependent magneto-resistance (MR) of Fe_1.5_Se_2_ at different temperatures. The arrows indicate the saturation magnetic fields of Fe_1.5_Se_2_. Red circles show the small resistance jumps, which may originate from the flip of magnetic domains. (d) Typical temperature-dependent *R*_xx_/*R*_300 K_ of Fe_1.6_Se_2_ nanoflake. (e) Magnetic field-dependent *R*_xy_ of Fe_1.6_Se_2_. The arrows indicate the saturation magnetic fields of Fe_1.6_Se_2_. (f) Field-dependent MR of Fe_1.6_Se_2_ at different temperatures. (g) *R*_xy_ and MR variation with magnetic field of Fe_1.5_Se_2_ nanoflake at 300 K. (h) *R*_xy_ and MR variation with magnetic field of Fe_1.6_Se_2_ nanoflake at 300 K. (i) In-plane MR of Fe_1.6_Se_2_ nanoflake at 10 K with magnetic field perpendicular to the *c* axis. Hall data were anti-symmetrically processed as a function of the magnetic field, and MR data were symmetrically processed.

Magneto-transport measurements were performed to explore the electrical behavior of Fe_1.5_Se_2_ and Fe_1.6_Se_2_. A typical Hall device is depicted in Fig. S19, and the applied magnetic field is perpendicular to the sample (Fig. 4a–h). Fig. 4a, d shows the variation of longitudinal resistance (*R*_xx_) from 390 to 5 K for Fe_1.5_Se_2_ and Fe_1.6_Se_2_, respectively, both of which decrease gradually with lowering temperatures. Their resistance data fits well with the phonon/magnon + gap scatterings model (Figs S20, S21) [27,36], indicating the half-metallic behavior of the two samples, which is identified with the following DFT results. Besides, their electrical conductivity is excellent compared with state-of-the-art 2D conductive materials (Table S1), showing their great potential as good 2D conductors.

Hall resistance (*R*_xy_) is displayed in Fig. 4b, e with smaller opening anomalous Hall effect (AHE), illustrating the in-plane magnetic ordering of Fe_1.5_Se_2_ and Fe_1.6_Se_2_ [37], in agreement with the magnetism characterizations. As the temperature decreases, the saturation magnetic fields (indicated by arrows) increase gradually and are not observed within 9 T below 100 K. Fig. 4c, f shows the magneto-resistance (MR) curves at different temperatures. When the magnetic field is larger than the saturation field, Fe_1.5_Se_2_ shows negative MR, because spin-dependent carrier scattering is suppressed. Notably, when it is below the saturation magnetic field, Fe_1.5_Se_2_ exhibits the crossover from negative MR to positive MR with decreasing temperatures (Fig. 4c and Fig. S22). This sign change may be ascribed to the transition from magnetism dominated MR (negative) to half-metallic nature dominated MR (positive) [27]. In a half-metal, electron-magnon scattering freezes out exponentially at low temperatures owing to the gapped minority spin states at the Fermi level, thus generating positive MR behavior [38,39]. As for Fe_1.6_Se_2_, negative MR is exhibited after exceeding the saturation magnetic field, while positive MR appears below the critical field in all temperature ranges (Fig. 4f and Fig. S23), which may result from the increased spin gap of Fe_1.6_Se_2_ than Fe_1.5_Se_2_. In addition to the resistance transition at the saturation field, there are also small jumps (highlighted by red circles) in both Hall and MR curves at the lower magnetic field (Figs S22, S23), which may be caused by the magnetic domains flipping from the in-plane direction (easy axis) to the out-of-plane magnetic field direction [2,40]. Besides, Fe_1.6_Se_2_ has more resistance steps than Fe_1.5_Se_2_, implying more complex domain patterns, which are identified with the MFM results. Moreover, MR transition behavior and AHE maintain up to 300 K (Fig. 4g, h), convincingly demonstrating the RT magnetism. More devices are fabricated to confirm the transport behavior (Figs S24, S25), and similar phenomena are observed. In-plane negative MR is observed in Fe_1.6_Se_2_ nanoflakes (Fig. 4i), demonstrating the in-plane magnetic ordering and the flipping of magnetic domain as the magnetic field increases.

**Figure 5.**
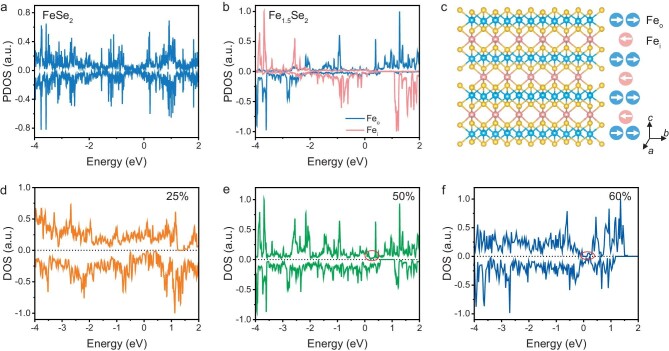
Calculated density of states in Fe_1+*x*_Se_2_. (a) Calculated spin-polarized projected density of states (PDOS) of Fe atoms in FeSe_2_. (b) PDOS of different Fe atoms in Fe_1.5_Se_2_. Fe_o_ represents original Fe atoms in the FeSe_2_ backbone and Fe_i_ denotes intercalated Fe atoms between FeSe_2_ gaps. (c) Schematic illustration of the spin structure in Fe_1.5_Se_2_. Blue balls represent Fe atoms in the FeSe_2_ backbone (Fe_o_), and red balls denote intercalated Fe atoms (Fe_i_). Yellow balls represent Se atoms. (d–f) Total DOS of Fe_1+*x*_Se_2_ when the intercalation ratio is 25% (d), 50% (e), and ∼60% (f), respectively. The Fermi level is set to be zero.

DFT calculations were further conducted to investigate the magnetic and electrical properties of Fe_1+*x*_Se_2_. FeSe_2_ is nonmagnetic due to the symmetry of density of states (DOS) between spin-up and spin-down electrons (Fig. 5a). However, the DOS symmetry of original Fe atoms (Fe_o_) in the FeSe_2_ backbone is broken by the intercalated Fe atoms (Fe_i_), as indicated in Fig. 5b, generating a magnetism moment of ∼3.24 *μ*_B_ per atom of Fe_o_ and about −3.37 *μ*_B_ per atom of Fe_i_ in Fe_1.5_Se_2_. Magnetic generation is attributed to the electron transfer between the FeSe_2_ backbone and intercalated Fe atoms, proved by the increase in the average Bader charge, as shown in Fig. S26. Their magnetic properties are solely contributed by the d-orbitals, because the symmetry in s- and p-orbitals cancels out their spin-up and spin-down contributions (Fig. S27). Therefore, the orderly intercalated Fe can induce a nonmagnetic structure to produce long-range magnetic coupling. The calculated magnetic moments are summarized in Table S2. Besides, the intralayer interactions are through a direct-exchange interaction and ∼90°Fe_o_-Se-Fe_o_ super-exchange interaction with ferromagnetic coupling [41]. Interlayer interactions are through a ∼128° and ∼69°Fe_o_-Se-Fe_i_ super-exchange interaction, favoring antiferromagnetic coupling [32,42]. Thus, the ferrimagnetic configuration is more energetically favored with 39.5 meV/atom lower than the ferromagnetic configuration in Fe_1.5_Se_2_. When increasing the IR, interlayer exchange integrals (*J*) are increased from 10.69 meV in Fe_1.5_Se_2_ to 34.40 meV in Fe_1.75_Se_2_, so the interlayer interaction between the FeSe_2_ backbone and Fe_i_ atom is significantly strengthened. Besides, intercalated atoms induce out-of-plane charge transfer (Fig. S26), resulting in modifications to the band structure. These alterations may change the magnetic anisotropy energy (MAE) and spin directions of the system [43,44]. Specifically, MAE changes from in-plane 32.99 μeV/atom in Fe_1.25_Se_2_ to out-of-plane 87.78 μeV/atom in Fe_1.75_Se_2_. As shown in Fig. S28, the calculated easy axis for Fe_1.25_Se_2_ and Fe_1.5_Se_2_ is along the *b*-axis, while the easy axis for Fe_1.75_Se_2_ is along the *c*-axis in its magnetic ground state, in accordance with the magnetic measurements. Spins are ferromagnetically arranged in the in-plane direction, while they show antiferromagnetically coupling along the out-of-plane direction. Notably, Fe_1.25_Se_2_ is a metallic material with DOS crossing the Fermi energy level (Fig. 5d), but the spin-up DOS of Fe_1.5_Se_2_ near the Fermi energy level approaches zero (Fig. 5e). A noteworthy observation is that Fe_1.6_Se_2_ has a larger band gap across the Fermi energy level in the spin-up channel, indicating an obvious half-metallic behavior (Fig. 5f). This transformation of band structures may be attributed to the upward shift of the Fermi level due to charge transfer and the enhanced orbital coupling between Fe and Se by increasing the IRs.

## CONCLUSION

In conclusion, the general rule for the relationship between IR and atomic structure in T phase self-intercalated TMD is summarized. Then, 2D synthesis of a family of Fe_1+*x*_Se_2_ nanoflakes with controllable IRs, including Fe_1.18_Se_2_ (disordered), Fe_1.25_Se_2_ Fe_1.5_Se_2_ (ordered), Fe_1.6_Se_2_ (half-ordered), and Fe_1.75_Se_2_ (ordered) is realized via a space confinement-assisted chemical potential regulation strategy. An innovative structure of ‘half-ordered intercalation’ is proposed, providing a new class of intriguing materials that may be discovered in other intercalated TMD. Aberration-corrected STEM characterizations verify the self-intercalation structures and the intercalation rule. Notably, intercalation can regulate both the magnetic and electrical properties (including Curie temperatures, spin directions, magnetic domains, spin gap, and MR). Disordered Fe_1.18_Se_2_ are nonmagnetic, while all orderly intercalated samples show RT magnetic ordering, which is induced by the charge transfer of intercalated Fe atoms. The magnetic structures transition from single- to multi-domain states with an increase in the IR. Strikingly, intercalation induces the generation of RT magnetic half-metals. Fe_1.5_Se_2_ shows a crossover from negative MR to positive MR below the saturation fields with decreasing temperatures, while Fe_1.6_Se_2_ keeps positive MR owing to the increased spin-up gap caused by more intercalated Fe atoms. Our work achieves the controllable synthesis of a new class of materials with remarkable properties, including obvious RT-AHE, spin-reorientation, and unique half-metallic behavior, which shows great promise in magnetic tunnel junctions and other spintronic devices. More importantly, we provide a classic paradigm for structural regulation of magnetic and electric properties.

## METHODS

### Synthesis of Fe_1+*x*_Se_2_ Nanoflakes by a space confinement-assisted chemical potential regulation strategy

The synthesis process was carried out in a three-temperature-zone tubular furnace. Se (Alfa Aesar, 99.5%) powder and H_2_ (10 sccm under the 100 sccm Ar as the carrier gas) were used to react with FeCl_2_ (Alfa Aesar, 99.5%); 50–600 mg Se powder was placed in the first heating zone, and 20–40 mg FeCl_2_ was placed in the second heating zone with the substrate on the top. Two freshly cleaved fluorophlogopite micas (Taiyuan Fluorphlogopite Mica Company Ltd, 10 × 10 × 0.2 mm) were stacked face-to-face, providing a confinement space to create a stable and uniform gas flow to precisely control the chemical potential during growth. The growth temperature (the second zone) is set at ∼580°C. The temperature of Se (the first zone) is changed from 300 to 500°C to regulate the mole fraction or chemical potential of Fe during the growth process and produce Fe_1+*x*_Se_2_. The key growth parameters for distinct IRs are shown in Table S3.

### Transfer of the Fe_1+*x*_Se_2_ nanoflakes

The mica covered by Fe_1+*x*_Se_2_ nanoflakes was spin-coated with poly(methyl methacrylate) (PMMA) film at a speed of 2000 r/min for 1 min once or twice, and baked at 120°C for 5 min. Afterwards, the edges of the PMMA film were scraped with a tweezer and immersed in a hydrofluoric acid solution to exfoliate the film from mica substrates, and then washed several times in deionized water. PMMA/Fe_1+*x*_Se_2_ film was further transferred onto the target substrates (Si/SiO_2_ or TEM grids), and baked at 110°C for ∼1 h in the glove box. In the end, acetone was employed to remove PMMA.

### Characterizations

An optical microscope (Nexcope NM910) was used to characterize the morphology and sizes of FeSe_*x*_ samples. Atomic force microscope and corresponding MFM modes (Bruker, Dimension Icon) were employed to analyze the thickness and magnetic phase images of 2D nanoflakes. XRD measurement (Rigaku DMAX-2400 X-ray diffractometer equipped with Cu Kα radiation) was used to illustrate the phase structure. Atomic-resolution STEM-ADF imaging was performed on an aberration-corrected JEOL ARM200F microscope operating at 200 kV. The convergence semiangle of the probe was ∼30 mrad. Image simulations were performed with the Prismatic package, assuming an aberration-free probe with a probe size of ∼1 Å. The convergence semiangle and accelerating voltage were in line with the experiments. The collection angle was between 80 and 200 mrad. STEM-ADF images were filtered by Gaussian filters, and the positions of atomic columns were located by finding the local maxima of the filtered series. EDS mapping (FEI, Tecnai F30) was performed to show the element compositions. Raman spectrum (Horiba, XploRA PLUS) with excitation light ∼532 nm was used. Magnetism was measured by the vibrating sample magnetometry modes in the physical property measurement system (PPMS) (DynaCool, Quantum Design) with the magnetic field up to 9 T and temperatures from 5 to 390 K. Hall data were anti-symmetrically processed as a function of the magnetic field, and MR data were symmetrically processed. Two-electrode testings were conducted on a probe station (Lakeshore TTP4) equipped with Keithley 4200 semiconductor analyzer. Magneto-transport measurements were performed by the electrical transport option (ETO) modes in the PPMS.

### DFT calculations

All the DFT calculations were performed using the Vienna *ab initio* simulation package (VASP) [45], where the electron-ion interaction of Fe and Se atoms were represented by the projector augmented wave (PAW) [46] scheme and the exchange-correlation functional utilized was GGA-PBE [47]. The kinetic energy cutoff was set to 600 eV. To estimate the effect of the strongly correlated *d* electron interactions, the GGA + *U* method was employed to take into account the strong correlation effect, with *U* = 2.0 eV for Fe. The calculation used the same atomic structure with experimental results. In order to account for the van der Waals (vdW) interaction, optPBE functional was used in the energy calculation [48]. The first Brillouin zone k-point sampling adopted the Monkhorst–Pack scheme with an automated mesh determined by 25 times the reciprocal lattice vectors. The initial structures were constructed by deleting Fe atoms in the intercalation sites of FeSe (*P*6_3_/*mmc*) superstructure with all intercalated Fe atoms positioned at the center of the octahedron. In order to obtain the most stable structural configuration, every composition was constructed with more than 15 different structures, containing both high-symmetry and randomly intercalated structures. Considering the intercalated Fe atoms would induce lattice distortion, here the symmetry was not preserved during structure optimization in order to achieve the most favorable configuration. For all the structures, both lattice and atomic positions were fully optimized until the maximal stress component was below 0.01 GPa, the maximal force component was below 0.05 eV Å^−1^, and the total energy difference was below 5 × 10^−6^ eV, respectively. The structure details of DFT calculations are shown in Fig. S29. Fe_1.18_Se_2_ and Fe_1.6_Se_2_ need to construct large crystal structures for DFT calculation, which is time-consuming and costly. Thus, we adopted the common IR of 0.167 (i.e. 1/6, just construct 2 × 3 × 2 supercells with 76 atoms) and 0.625 (i.e. 5/8, just construct 2 × 2 × 2 supercells with 58 atoms) in DFT calculations, which was close to 0.18 and 0.6 but can reduce the computation time, resulting in cost savings. Importantly, the concentration difference is only ∼0.02, which has little effect on the analysis by DFT calculations.

## Supplementary Material

nwae430_Supplemental_File
