# Supplementary material for: The evolution of chemical ordering and property in Fe1+xSe2 upon intercalation ratios
Source: Natl Sci Rev. 2024 Nov 29;12(2):nwae430. doi: 10.1093/nsr/nwae430 (PMC11737390; doi:10.1093/nsr/nwae430)
Supplement: nwae430_Supplemental_File [file nwae430_supplemental_file.pdf]

Supplementary Information

The evolution of chemical ordering and property in $Fe_{1+x}Se_2$ upon intercalation ratios

Zijing Zhao[1,2,3], Xiaocang Han[1], Shengcai Zhu[2], Zhi Fang[1], Ziyi Han[1], Zhongyu Liang[4], Bailing Li[1], Biao Zhang[1], Wei Li[1], Zhaochu Luo[4], Licong Peng[1], Xiaoxu Zhao[1]*, Xiangguo Li[2], Jiadong Zhou[5], Song Gao[6], Chengxin Wang[7], Mathias Kläui,[8] Yanglong Hou[1,2]*

[1]School of Materials Science and Engineering, Beijing Key Laboratory for Magnetoelectric Materials and Devices, Peking University, Beijing 100871, China.

[2]School of Materials, Shenzhen Campus of Sun Yat-sen University, Shenzhen 518107, China.

[3]School of Physics and Optoelectronic Engineering, Beijing University of Technology, Beijing 100124, China.

[4]State Key Laboratory for Artificial Microstructure & Mesoscopic Physics, School of Physics, Peking University, Beijing 100871, China.

[5]Key Lab of Advanced Optoelectronic Quantum Architecture and Measurement (MOE), Beijing Key Lab of Nanophotonics & Ultrafine Optoelectronic Systems, and School of Physics, Beijing Institute of Technology, Beijing 100081, China

[6]School of Chemistry, Sun Yat-Sen University, Guangzhou 510275, China

[7]School of Materials Science and Engineering, Sun Yat-Sen University, Guangzhou 510275, China

[8]Institute of Physics, Johannes Gutenberg University Mainz, Mainz 55128, Germany

*Corresponding author. E-mails: xiaoxuzhao@pku.edu.cn; hou@pku.edu.cn

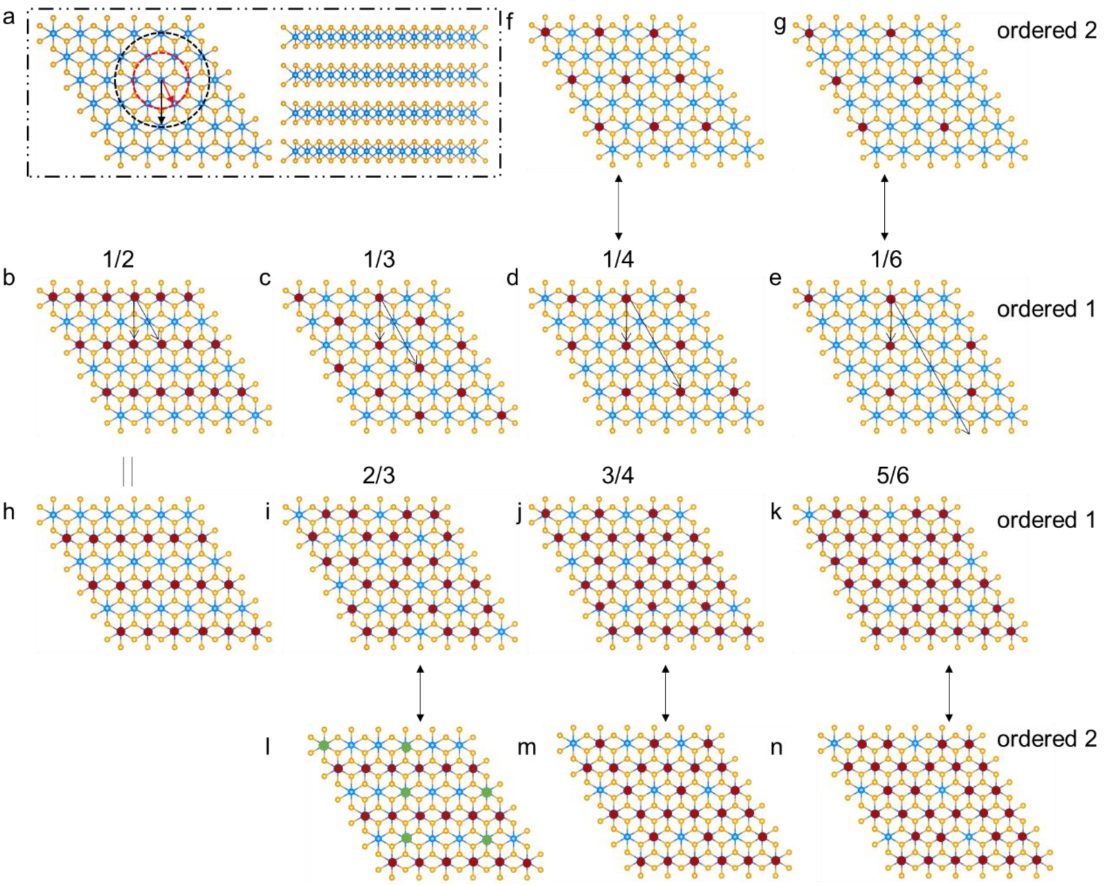

Figure S1. Intercalated structures of $Fe_{1+x}Se_2$ with various ratios. a) Atomic structure of T phase $FeSe_2$ backbone. Blue balls denote Fe atoms, and yellow balls denote Se atoms. The red circle shows the distribution of the nearest Fe atoms, and the black circle shows the distribution of the next nearest Fe atoms. b-n) Intercalation sites of 1/N (i.e. 1/2, 1/3, 1/4, 1/6) and (N-1)/N (i.e. 2/3, 3/4, 5/6) Fe-intercalated $FeSe_2$ indicated in the *ab* plane, respectively. (f, g, l, m, n) show the rearrangement of intercalated sites (labeled by ordered 2). Red and green balls indicate the intercalated sites.

On the 2D $FeSe_2$ basal plane, the first nearest Fe-Fe distance is about 3.80 Å, and forms a close packing line (as the red circle in Figure S1a); while the second nearest Fe-Fe distance is 6.59 Å ($\sqrt{3}a$), forming a line 30 degrees depart from the close packing line (shown in black circles).

Intercalation atoms prefer to form supercells along the nearest metal sites due to the largest interaction, and the structures with higher symmetry are more stable. In addition, (N-1)/N intercalation can also be viewed as full interaction at one kind of site (red balls in Figure S1l) and ordered interaction at another kind of site (green balls). Figure S1 gives almost all ordered arrays of intercalation states.

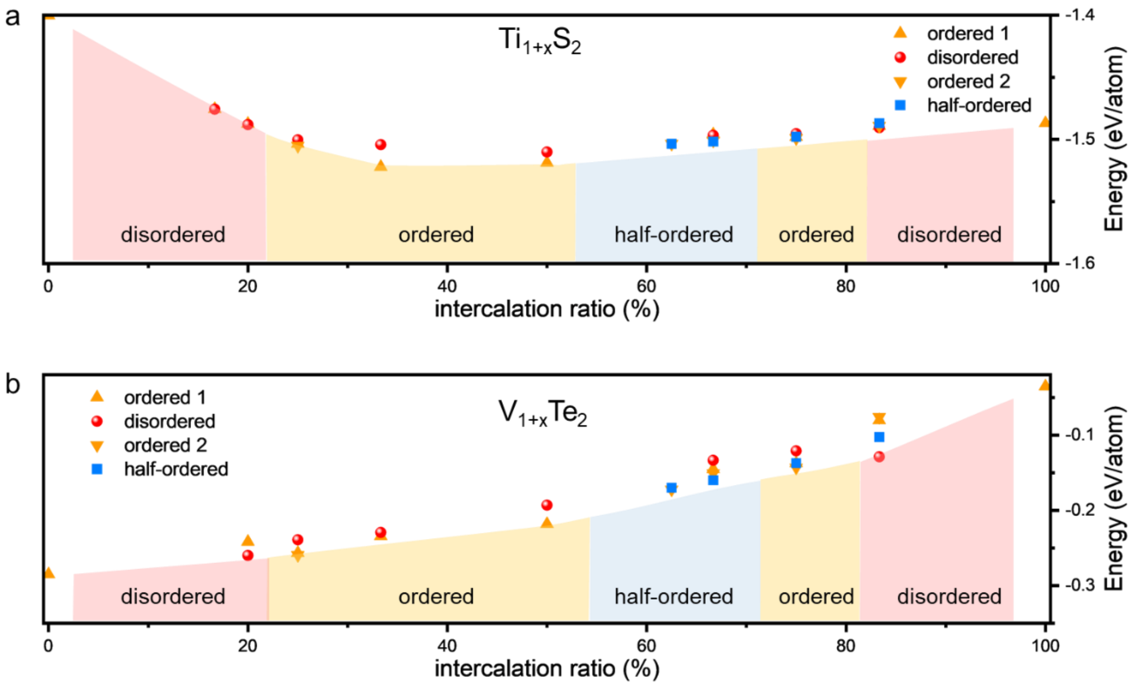

Figure S2. The formation energy of $Ti_{1+x}S_2$ and $V_{1+x}Te_2$ (T phase) with various intercalation ratios in different intercalated structures.

$Ti_{1+x}Se_2$ and $V_{1+x}Te_2$ have similar intercalation rule: the thermodynamically stable intercalation structure changes from disordered, to ordered, then to half-ordered (IR>0.5), back to ordered, and further to disordered structure, with increasing the intercalation ratio.

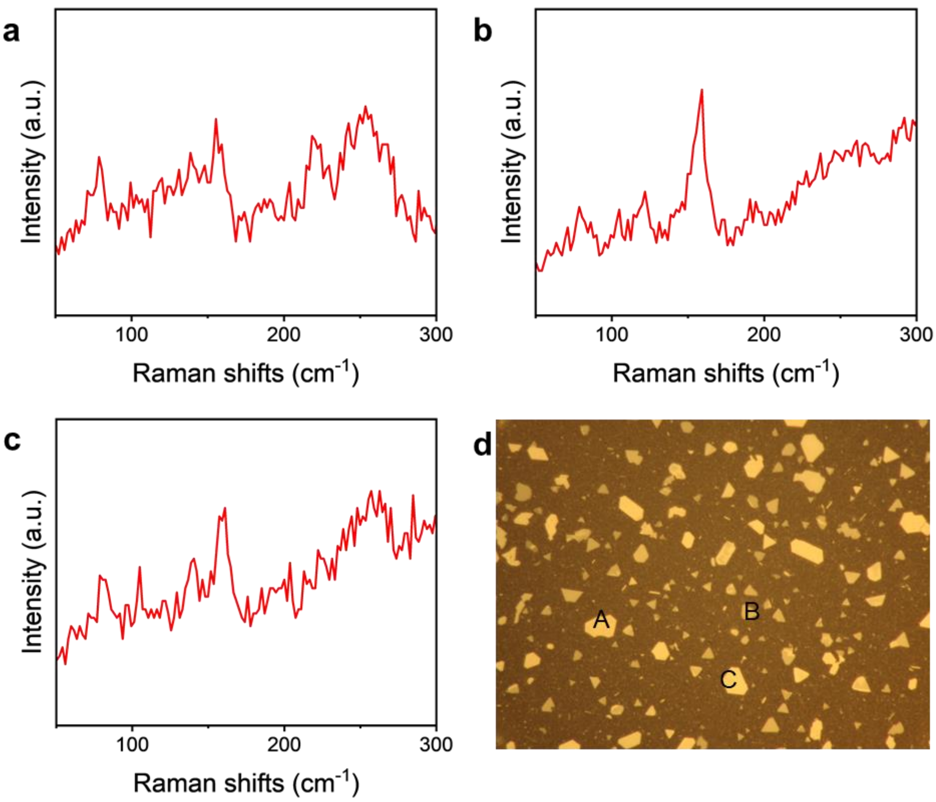

Figure S3. The optical microscope (OM) image and corresponding Raman spectra of the samples on mica in the non-confined space. a) Raman spectrum of the sample-A labeled in (d). b) Raman spectrum of the sample-B labeled in (d). c) Raman spectrum of the sample-C labeled in (d). d) The OM image of the samples in the non-confined space.

Turbulent flow will occur in the non-confined space, which makes the concentration distribution of the precursor uneven during growth, bringing significant challenges in controlling the chemical potential. As is shown in Figure S3, the Raman spectra of the samples synthesized in the non-confined space are different (A is $Fe_{1.6}Se_2$, B is $Fe_{1.18}Se_2$, and C is $Fe_{1.5}Se_2$), so it is not conducive to preparing pure phases. However, the confined space reduces the density of reactants and gas flow rate to create a more stable laminar gas transport, providing uniform chemical potential in the growth process. Controllably regulating the chemical potential of metals can facilitate the synthesis of various $Fe_{1+x}Se_2$ nanoflakes with pure phases.

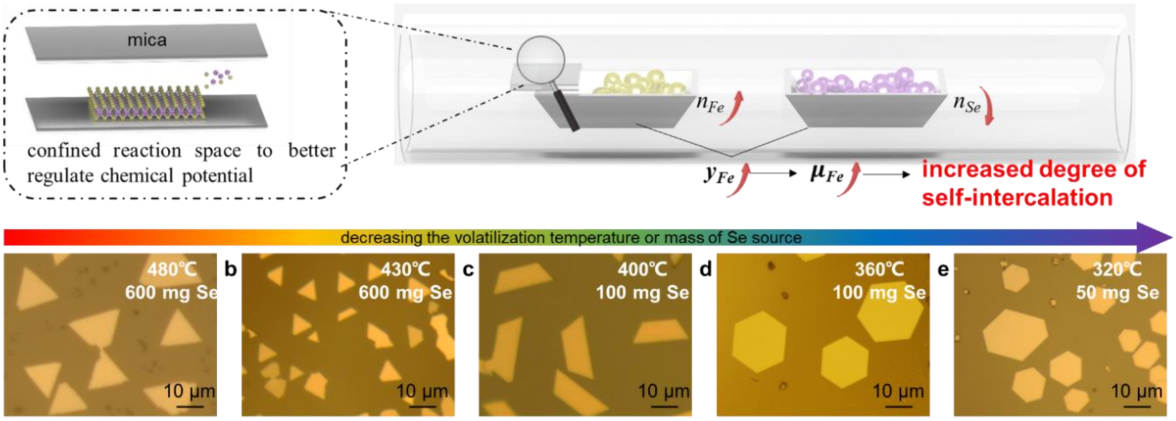

Figure S4. Controllable synthesis of 2D $Fe_{1+x}Se_2$ nanoflakes by regulating the chemical potential. a) OM image of $Fe_{1.18}Se_2$ nanoflakes with triangles. b) OM image of $Fe_{1.25}Se_2$ nanoflakes with mostly triangles. c) OM image of $Fe_{1.5}Se_2$ nanoflakes with semi-hexagons. d) OM image of high Fe-intercalated $Fe_{1.6}Se_2$ nanoflakes with hexagons. e) OM image of higher Fe-intercalated $Fe_{1.75}Se_2$ nanoflakes.

By decreasing the volatilization temperature or mass of Se source and keeping the other growth conditions unchanged, the concentration of Se vapor (n_{Se}) in the CVD tube is reduced. Simultaneously, the volatility of $FeCl_2$ precursor is improved (the surface of $FeCl_2$ precursor is less poisoned by Se), so the concentration of Fe vapor (n_{Fe}) is increased. Therefore, the molar fraction of Fe (y_{Fe}) in the CVD tube, *i.e.* the ratio of the moles of Fe to the total moles of all components (there are only Fe- and Se-related compounds), is greatly increased. Additionally, chemical potential is expressed as $\mu(T,P)=\mu^*(T,P)+RTlny$, where μ^*_{Fe} remains a constant because the growth temperature (T) is fixed and the pressure (P) is under atmospheric pressure, R is the molar gas constant, and y_{Fe} is now elevated. As a result, the chemical potential of Fe (μ_{Fe}) is augmented.

With increasing the chemical potential of Fe, intercalation ratios are enlarged. Meanwhile, the nanoflakes exhibit the transition from triangular to hexagonal shapes, which may be related to the difference of growth rate between metal-terminated and chalcogenide-terminated edges[1].

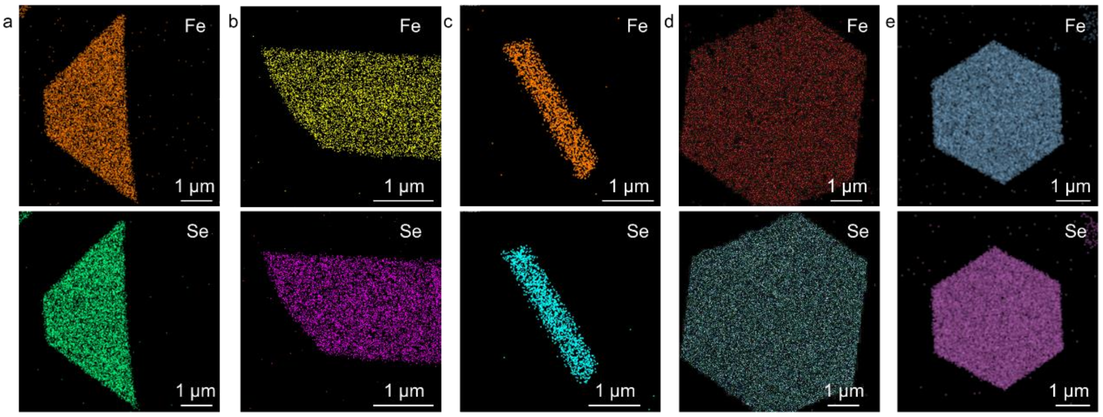

Se: Fe	materials
1.76	a
1.45	b
1.31	c
1.24	d
1.13	e

Figure S5. Energy dispersive spectroscopy (EDS) measurement of $Fe_{1+x}Se_2$ nanoflakes. a) EDS elemental mapping images of Fe and Se element in $Fe_{1.18}Se_2$. b) EDS elemental mapping images of Fe and Se element in $Fe_{1.25}Se_2$. c) EDS elemental mapping images of Fe and Se element in $Fe_{1.5}Se_2$. d) EDS elemental mapping images of Fe and Se element in $Fe_{1.6}Se_2$. e) EDS elemental mapping images of Fe and Se element in $Fe_{1.75}Se_2$.

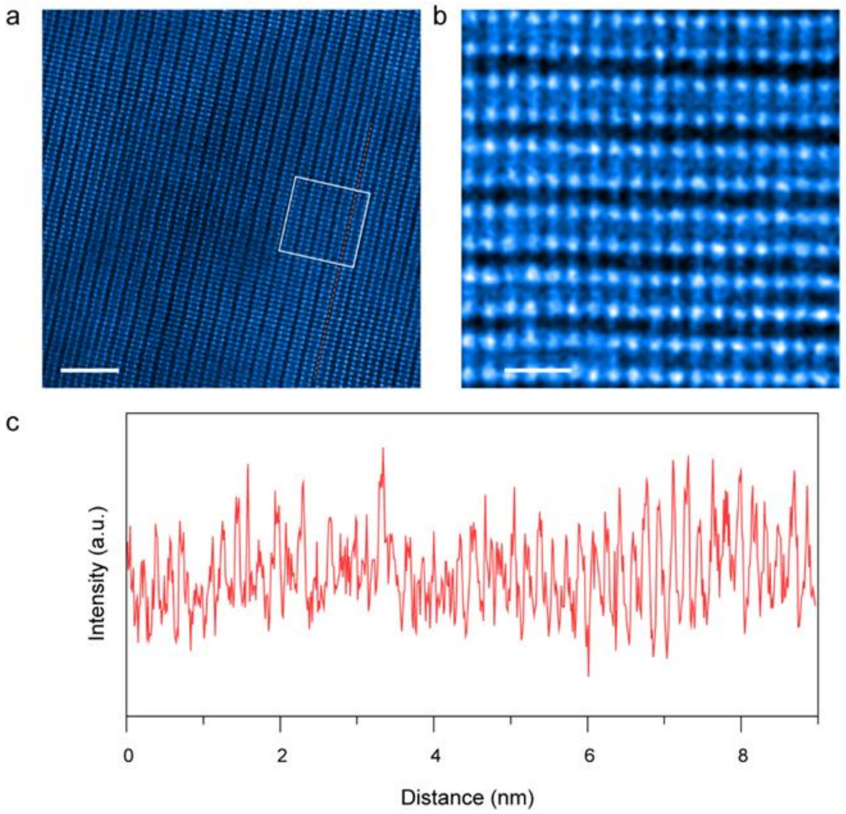

Figure S6. Side-view STEM image of $Fe_{1.18}Se_2$. a) Cross-sectional STEM image of $Fe_{1.18}Se_2$. b) Zoom-in STEM image from white box in (a). c) The intensity line profiles from red line in (a). Scale bars: 2 nm in (a); 0.5 nm in (b).

Side-view STEM images of $Fe_{1.18}Se_2$ exhibit the random distributions of intercalated Fe atoms (non-uniform) in the vdW gaps of $Fe_{1.18}Se_2$.

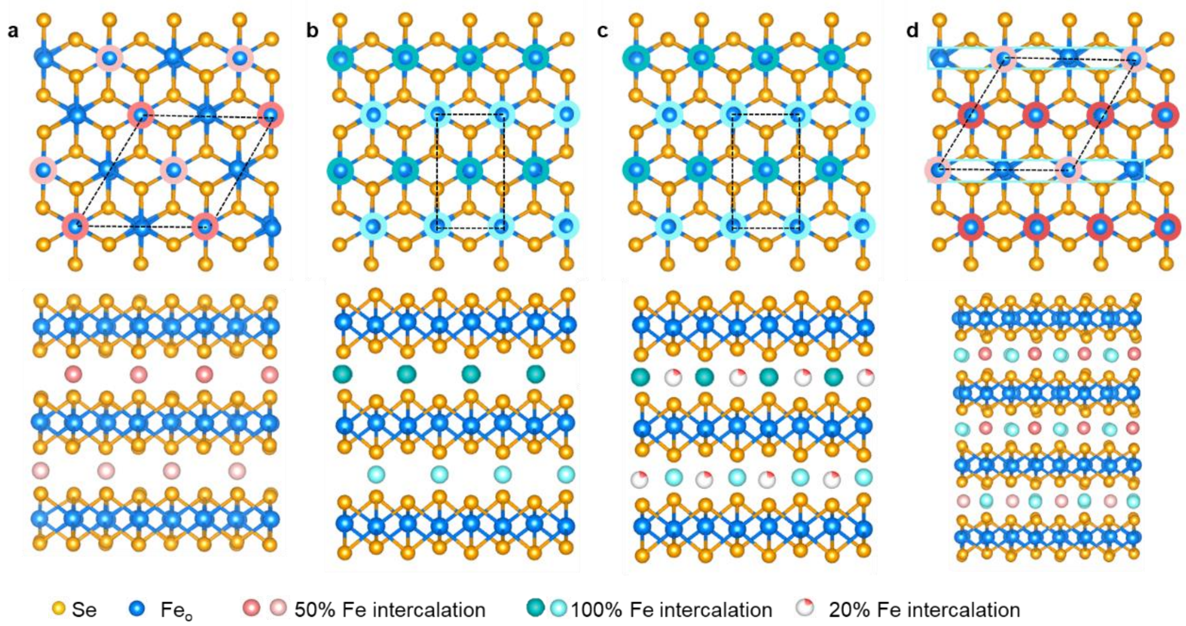

Figure S7. Structure models of $Fe_{1+x}Se_2$. a) Structure models of $Fe_{1.25}Se_2$ (25% intercalation) with rhombic symmetry. b) Structure models of $Fe_{1.5}Se_2$ (50% intercalation) with rectangular symmetry. c) Structure models of $Fe_{1.6}Se_2$ (60% intercalation) with rectangular symmetry. d) Structure models of $Fe_{1.75}Se_2$ (75% intercalation) with rhombic symmetry.

$Fe_{1.25}Se_2$ has 50% intercalation (filling every second vacancy) at half sites **(ordered)**.

$Fe_{1.5}Se_2$ has 100% intercalation at half sites **(ordered)**.

In $Fe_{1.6}Se_2$, one kind of intercalated sites is the same as $Fe_{1.5}Se_2$ with 100% occupation **(ordered)**, but the other kind of sites is 20% occupation **(disordered)**.

In $Fe_{1.75}Se_2$, 100% intercalation at one kind of sites **(ordered)**, and 50% intercalation at another kind of sites like $Fe_{1.25}Se_2$ **(ordered)**.

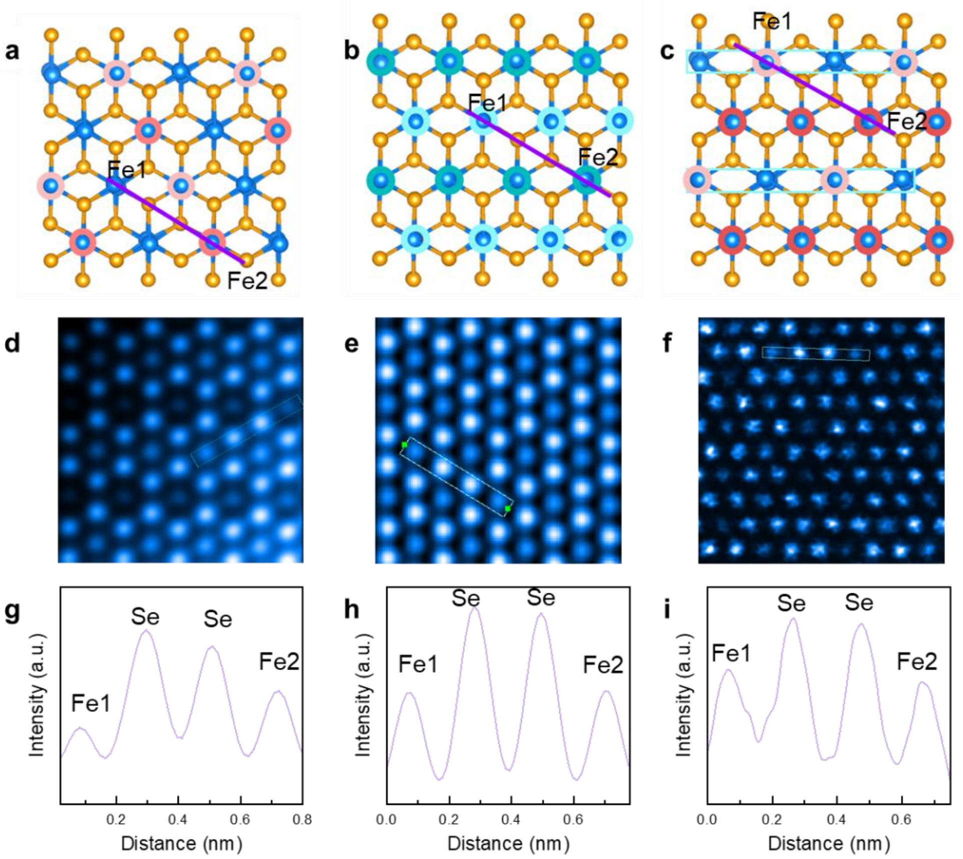

Figure S8. Top-view STEM analysis. a-c) Structure models along the *c* axis of $Fe_{1.25}Se_2$ (a), $Fe_{1.5}Se_2$ (b), and $Fe_{1.75}Se_2$ (c), respectively. d-f) Top-view atomic-resolution STEM images of 2D $Fe_{1+x}Se_2$. g-i) Intensity line profiles along the highlighted lines inserted in (d-f).

The periodicity (Fig. S8d-f) and intensity variations of the Fe-Se-Se-Fe atom (Fig. S8g-i) in top-view images are consistent with the construct model (Fig. S8a-c), demonstrating the veracity and accuracy of the model.

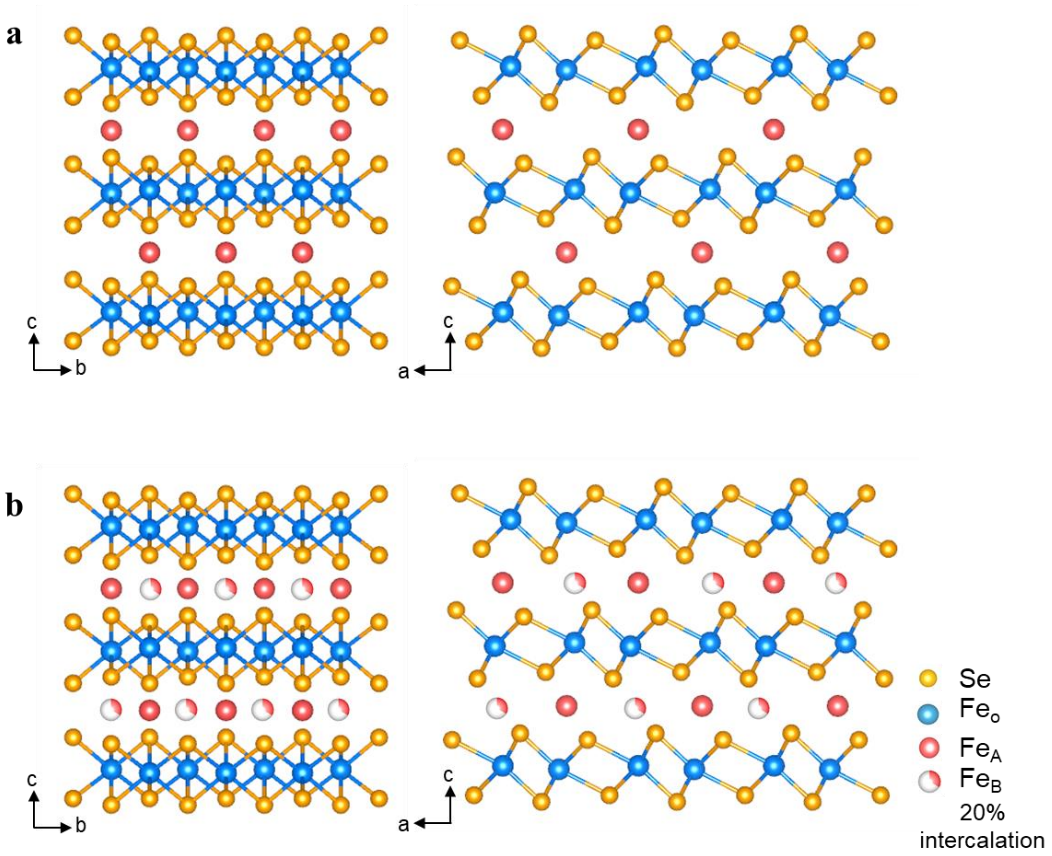

Figure S9. Atomic structures of $Fe_{1.6}Se_2$ compared with $Fe_{1.5}Se_2$. a) Atomic model of $Fe_{1.5}Se_2$ along the *a* axis and *b* axis, respectively. b) Atomic model of $Fe_{1.6}Se_2$ along the *a* axis and *b* axis, respectively.

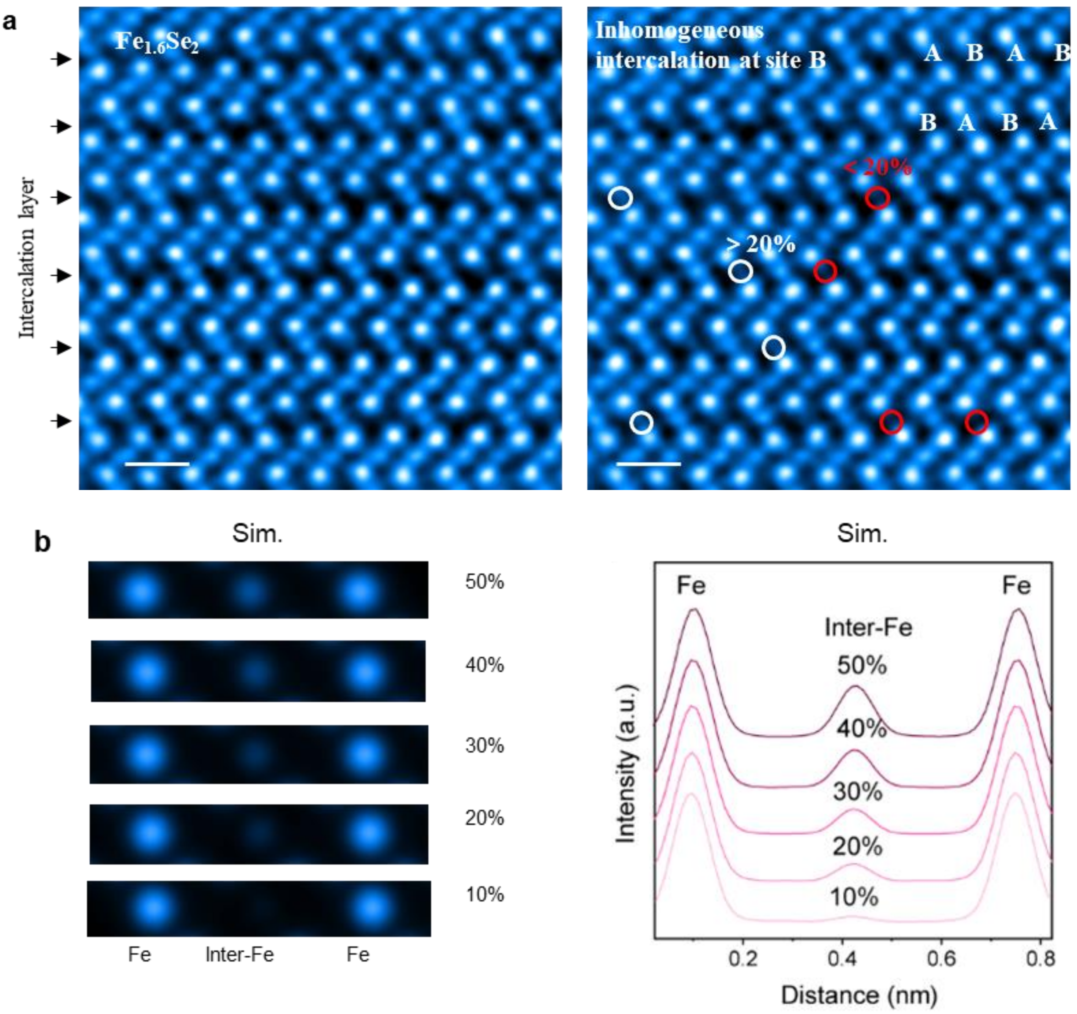

Figure S10. STEM images of $Fe_{1.6}Se_2$. a) The inhomogeneous intercalation at sites B of $Fe_{1.6}Se_2$. b) Simulated STEM images and corresponding intensity line profiles of different intercalation ratios.

Fe_A and Fe_B sites indicate two types of intercalated sites in the vdW gaps of $FeSe_2$ backbone. In $Fe_{1.6}Se_2$, site A is orderly intercalated with Fe atoms (100% intercalation), but the intercalated Fe atoms in site B are disordered and inhomogeneous at the atomic scale (Fig. S10a).

Figure 2e is the averaged STEM image of $Fe_{1.6}Se_2$ to estimate its intercalation concentration. Fe_B sites have averaged ~20% intercalation verified by the consistency of intensity line profiles in the simulated and experimental images shown in Fig. S10b and Figure 2o.

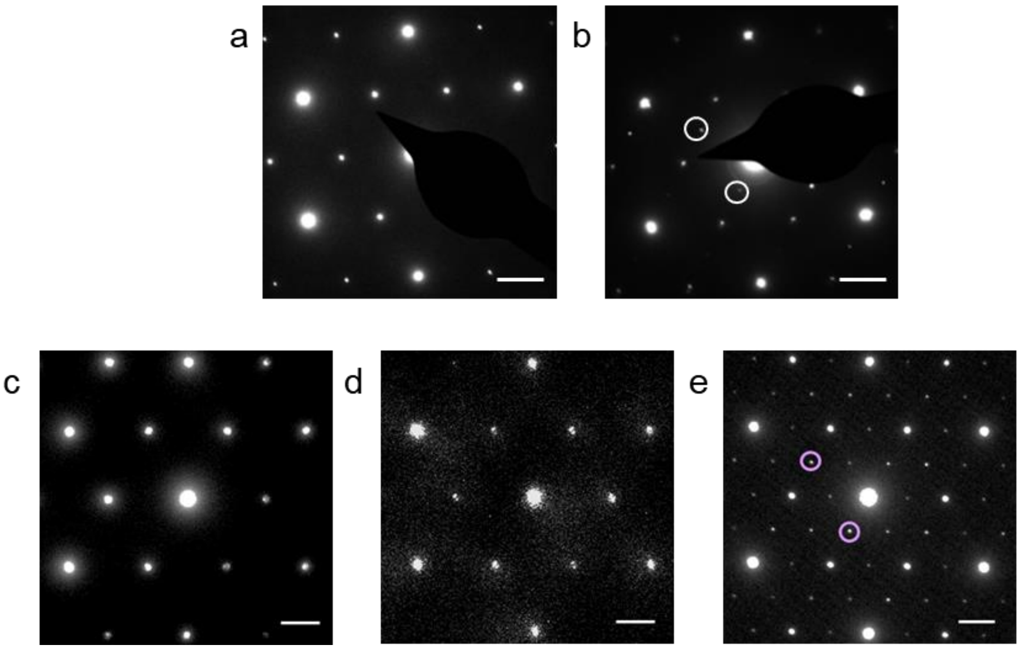

Figure S11. Corresponding SAED patterns of $Fe_{1.18}Se_2$ (a), $Fe_{1.25}Se_2$ (b), $Fe_{1.5}Se_2$ (c), $Fe_{1.6}Se_2$ (d), $Fe_{1.75}Se_2$ (e), respectively. Scale bar: 2 1/nm.

White or purple circles indicate the superspots originate from the ordered arrangement of intercalated Fe atoms.

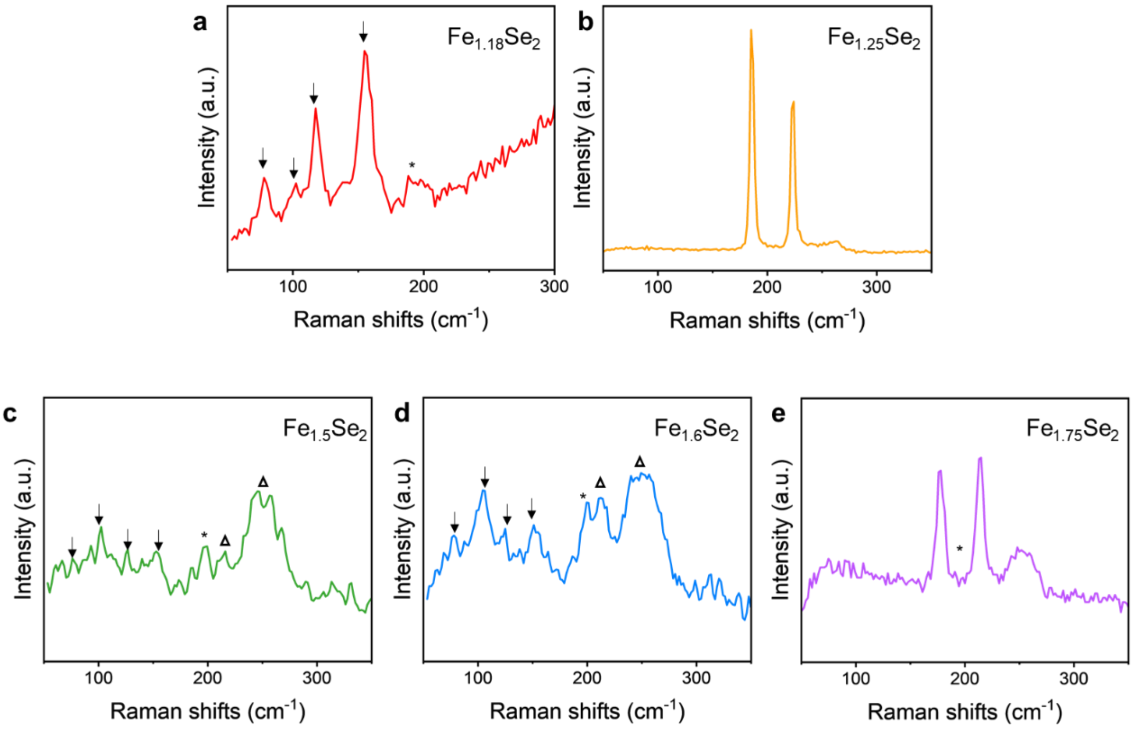

Figure S12. Raman spectrum of $Fe_{1.18}Se_2$, $Fe_{1.25}Se_2$, $Fe_{1.5}Se_2$, $Fe_{1.6}Se_2$, $Fe_{1.75}Se_2$, respectively. * indicates the Raman peak of mica substrate.

The Raman peaks of $Fe_{1.18}Se_2$ are located at ~78 cm^{-1}, ~102 cm^{-1}, ~117 cm^{-1}, and ~154 cm^{-1}, respectively, which may originate from the vibration of $FeSe_2$ backbone[2]. The peaks of $Fe_{1.25}Se_2$ are positioned at ~185 cm^{-1} and ~224 cm^{-1}. The peaks of $Fe_{1.5}Se_2$ and $Fe_{1.6}Se_2$ in the range from 50 cm^{-1} to 200 cm^{-1} are at similar positions with $FeSe_2$ backbone (indicated by arrows), but have weak intensities. Besides, $Fe_{1.5}Se_2$ and $Fe_{1.6}Se_2$ possess two additional Raman peaks at ~212 cm^{-1} and ~250 cm^{-1} (indicated by triangles). Moreover, the Raman peaks of $Fe_{1.75}Se_2$ appear at 178 cm^{-1}, ~215 cm^{-1}, and ~250 cm^{-1}.

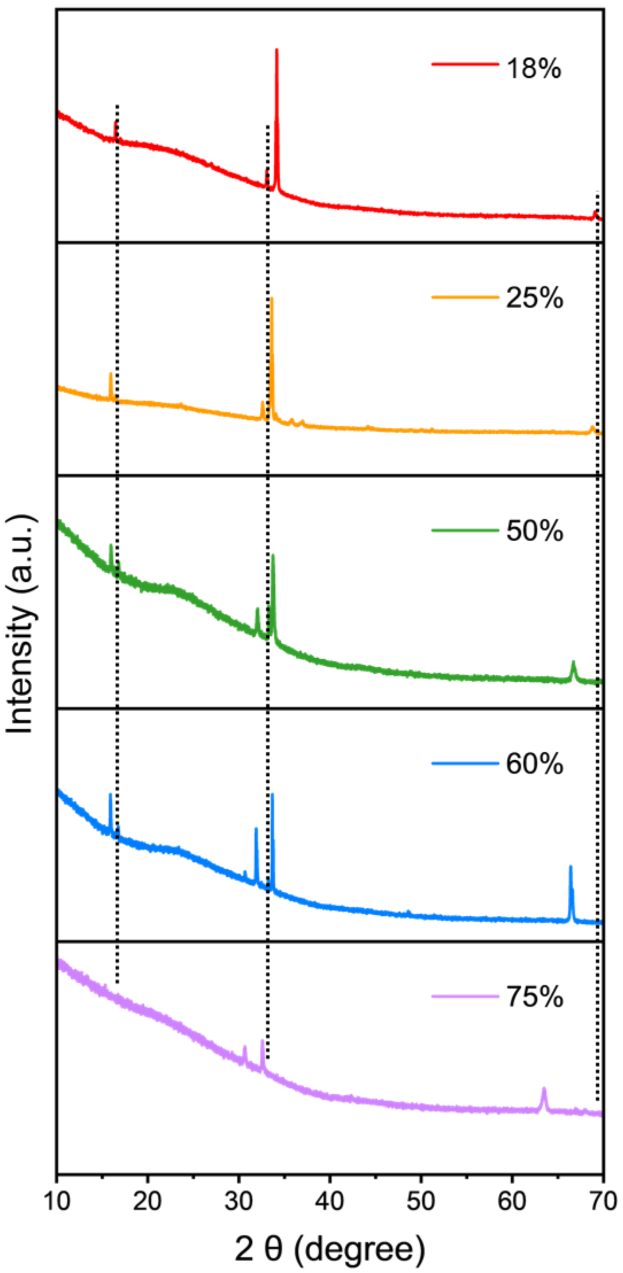

Figure S13. X-ray diffraction (XRD) patterns of $Fe_{1+x}Se_2$.

As the intercalation concentration increases, the peaks shift towards lower XRD angles, indicating that the interlayer gaps gradually increase, which is consistent with the STEM result (the interlay gap increases from ~5.3 Å in $Fe_{1.18}Se_2$ to ~5.9 Å in $Fe_{1.75}Se_2$). Besides, XRD is a reliable method to validate the purity of samples in a large area. The absence of any impure peaks in the XRD spectra of these samples demonstrates their pure phases.

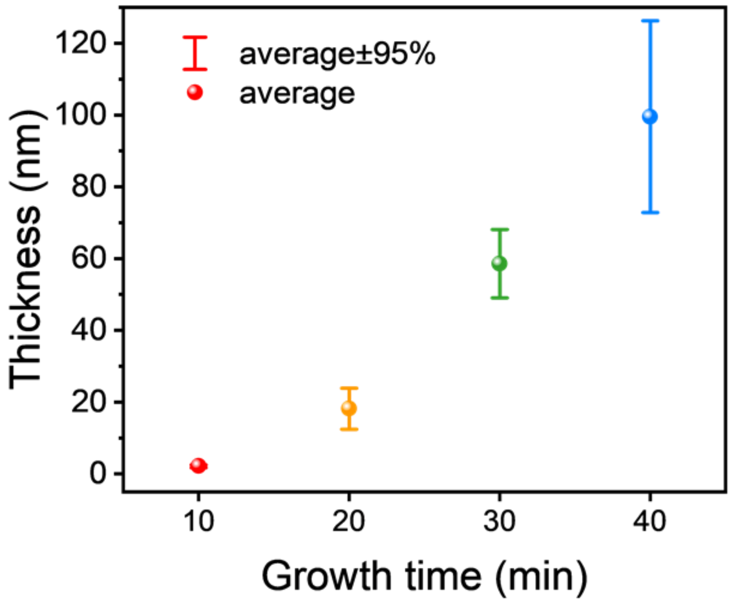

Figure S14. The thickness of $Fe_{1.6}Se_2$ can be regulated via adjusting the growth time.

With increasing the growth time, the average thickness of Fe-intercalated samples ($Fe_{1.6}Se_2$) increases gradually from ~2.1 nm (10 min) to ~99.8 nm (40 min).

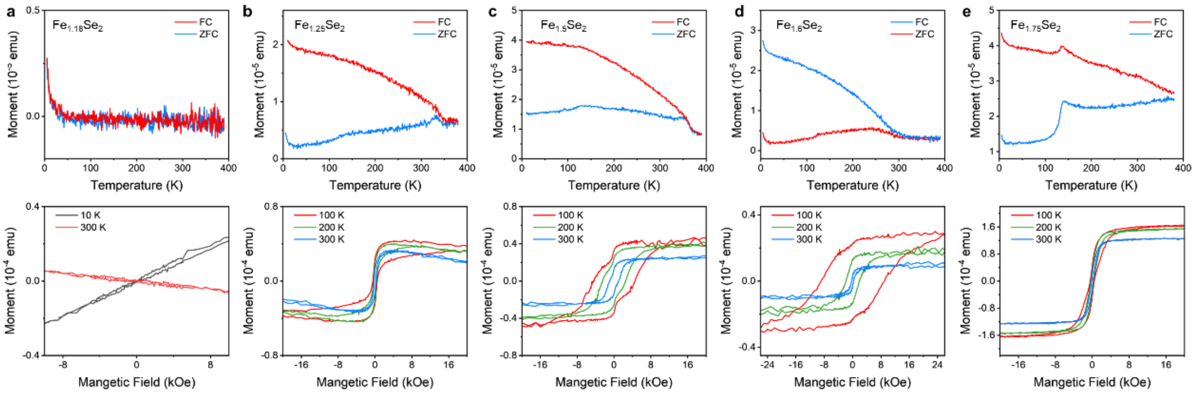

Figure S15. Magnetization curves of $Fe_{1+x}Se_2$. a) In-plane field-cooled (FC) and zero-field-cooled (ZFC) magnetization curves of $Fe_{1.18}Se_2$ with the magnetic field of 500 Oe, and the magnetization versus magnetic field curves of $Fe_{1.18}Se_2$ at 10 K and 300 K, respectively. b) In-plane FC and ZFC magnetization curves of $Fe_{1.25}Se_2$ with the magnetic field of 500 Oe, and the magnetization versus magnetic field curves of $Fe_{1.25}Se_2$ at 100 K, 200 K, and 300 K, respectively. c) In-plane FC and ZFC magnetization curves of $Fe_{1.5}Se_2$ with the magnetic field of 500 Oe, and the magnetization versus magnetic field curves of $Fe_{1.5}Se_2$ at 100 K, 200 K, and 300 K, respectively. d) In-plane FC and ZFC magnetization curves of $Fe_{1.6}Se_2$ with the magnetic field of 500 Oe, and the magnetization versus magnetic field curves of $Fe_{1.6}Se_2$ at 100 K, 200 K, and 300 K, respectively. e) In-plane FC and ZFC magnetization curves of $Fe_{1.75}Se_2$ with the magnetic field of 500 Oe, and the magnetization versus magnetic field curves of $Fe_{1.75}Se_2$ at 100 K, 200 K, and 300 K, respectively.

As shown in Figure S15a, the FC curve overlaps with ZFC curve, and there is no hysteresis phenomenon even at 10 K, indicating that $Fe_{1.18}Se_2$ does not have long-range magnetic ordering. However, magnetization curves of high intercalated $Fe_{1+x}Se_2$ show ferrimagnetic coupling.

The Curie temperature of $Fe_{1.25}Se_2$, $Fe_{1.5}Se_2$, and $Fe_{1.6}Se_2$ is around ~300 K. $Fe_{1.5}Se_2$ and $Fe_{1.6}Se_2$ have large coercivity (with about ~2 kOe in the in-plane direction at 200 K) due to the uniaxial magnetocrystalline anisotropy of its monoclinic structure[3], which are much larger than previous 2D materials[4-9].

However, the ZFC and FC magnetization curves of $Fe_{1.75}Se_2$ do not overlap even at 390 K, showing that its Curie temperature is higher. Moreover, there is a transition in the ZFC and FC curves of $Fe_{1.75}Se_2$, indicating a unique spin reorientation behavior at ~130 K[10].

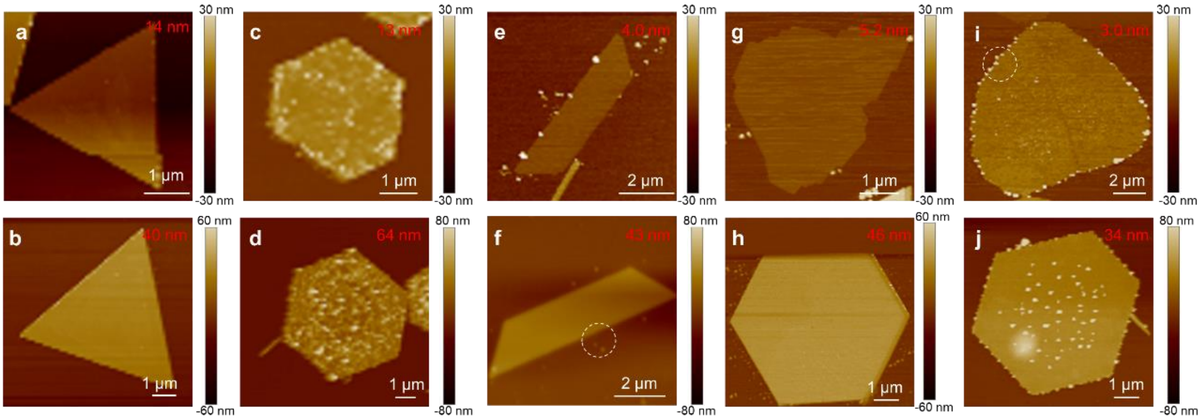

Figure S16. Corresponding topography images of the MFM phase images in Figure 3f-j.

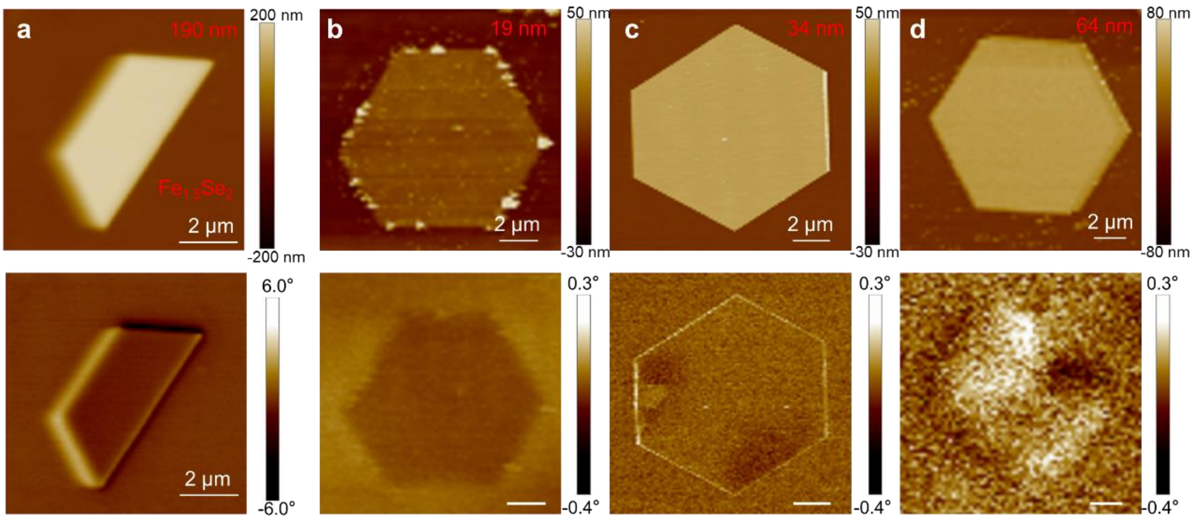

Figure S17. Supplementary MFM images of $Fe_{1+x}Se_2$. a) MFM phase image and corresponding topography image of $Fe_{1.5}Se_2$ with the thicknesses of ~190 nm. b-d) MFM phase image and corresponding topography image of $Fe_{1.6}Se_2$ with different thicknesses.

$Fe_{1.5}Se_2$ nanoflakes are in single-domain states even at ~190 nm (Figure S17a), while thinner $Fe_{1.6}Se_2$ nanoflakes are in single-domain states but thicker $Fe_{1.6}Se_2$ nanoflakes are in multi-domain states (Figure S17b-d).

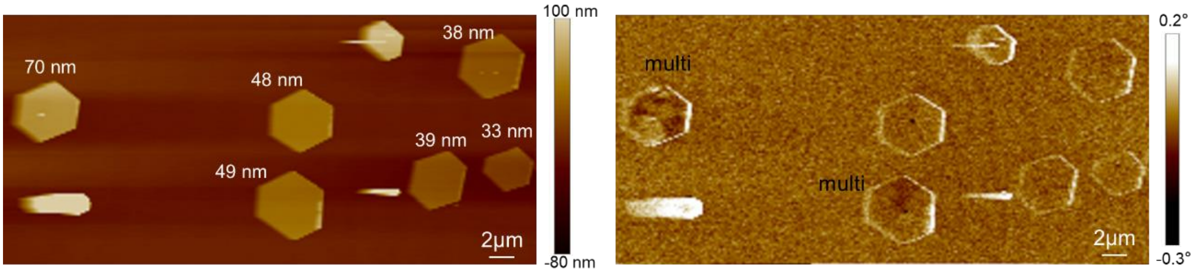

Figure S18. MFM phase images and corresponding topography images of $Fe_{1.6}Se_2$ nanoflakes with different thicknesses.

As is shown in Figure S18, multi-domain magnetic structures are displayed in thicker samples (>49 nm), while single-domain states are exhibited in thinner ones. The thickness-dependent magnetic states originate from the decrease of magnetostatic energy by decreasing the thickness which cannot compensate for the increasement of domain wall energy[11-13].

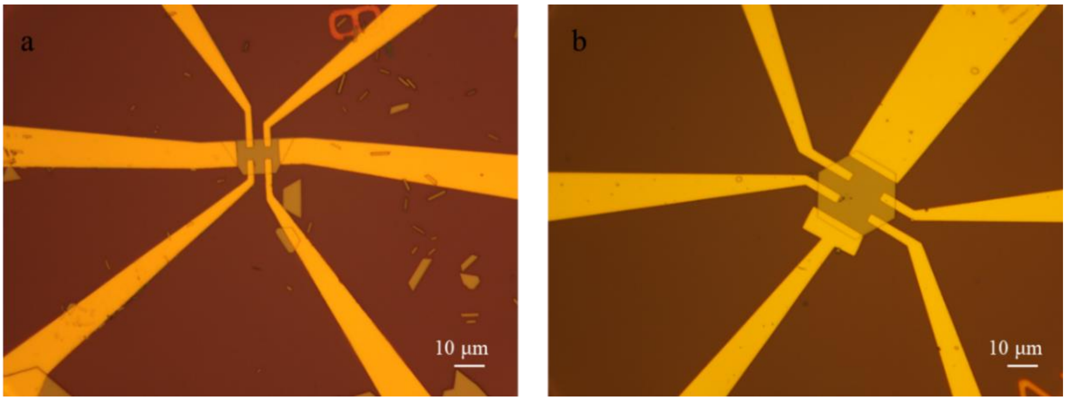

Figure S19. OM images of Hall devices based on $Fe_{1.5}Se_2$ (a) and $Fe_{1.6}Se_2$ (b).

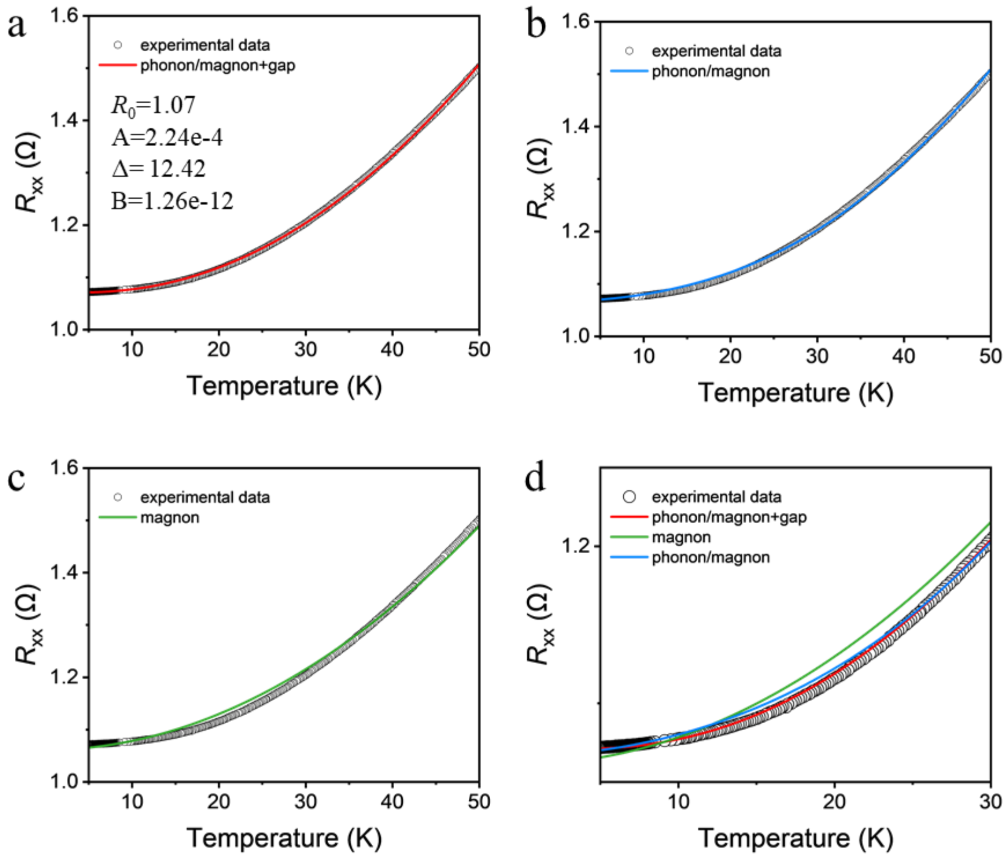

Figure S20. The longitudinal resistance (R_{xx}) versus temperature curves of $Fe_{1.5}Se_2$ fitted by different models. a) The fitted curve using the phonon/magnon +gap model. The fitting parameters are shown as well. b) The fitted curve using the phonon/magnon model. c) The fitted curve using the magnon model. d) The comparison of three models, where the phonon/magnon +gap model fits best.

Low-temperature resistance was analyzed to understand the transport mechanism in $Fe_{1+x}Se_2$ samples. As for the magnetic metal system, resistance (R) can be expressed as follows[14-15]:

$$R(T)= R_0+R_M(T)+ R_P(T)$$

$$R_M(T) = AT^2e^{(-\frac{\Delta}{T})}$$

$$R_P(T)= B(\frac{T}{\theta_D})^5 \int_0^{\frac{\theta_D}{T}} \frac{x^5}{(e^x-1)(1-e^{-x})}dx$$

where R_0 is the temperature-independent term (caused by defects in crystal lattice), R_M is the magnonic term, and R_P is the phononic term.

A represents magnon scattering strength. T represents temperatures. Δ measures the energy gap at the Fermin energy level (E_F) of unoccupied minority spins in half metals, which equals 0 in metals. B denotes phonon scattering exponent and θ_D is Debye temperature (θ_D is estimated to be ~262 K in Fe_3Se_4[16]).

Notably, R does not fit well with the pure magnon [$R(T)= R_0+AT^2$] nor phonon/magnon scatterings [$R(T)= R_0+AT^2+R_P(T)$]. Therefore, the conventional electron-magnon scattering of ferromagnets cannot explain the observed temperature-dependent resistance. If considering the half-metallic characteristic, the phonon/magnon+gap scatterings [$R(T)= R_0+AT^2e^{(-\frac{\Delta}{T})}+R_P(T)$] fit well. In other words, $R(T)$ follows an exponentially suppression of spin-flip scattering, indicating the half-metallic nature of $Fe_{1.5}Se_2$.

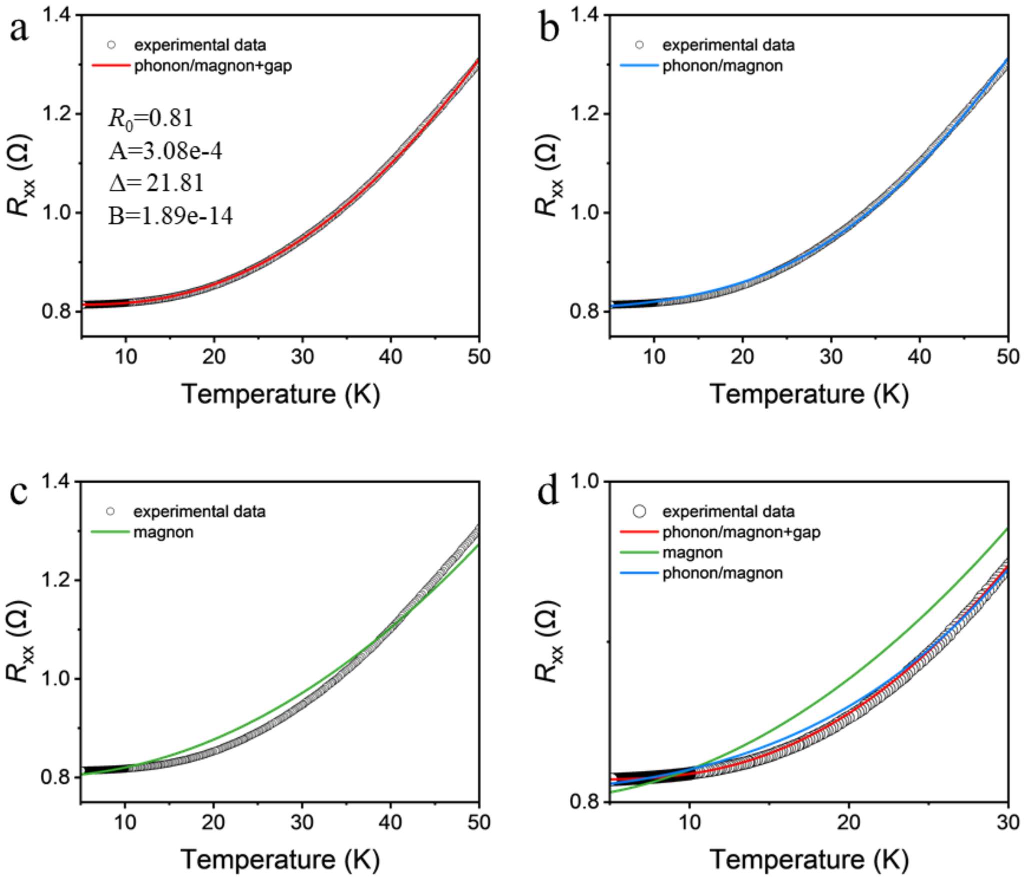

Figure S21. R_{xx} versus temperature curves of $Fe_{1.6}Se_2$ fitted by different models. a) The fitted curve using the phonon/magnon +gap model. The fitting parameters are shown as well. b) The fitted curve using the phonon/magnon model. c) The fitted curve using the magnon model. d) The comparison of three models, where the phonon/magnon +gap model fits best.

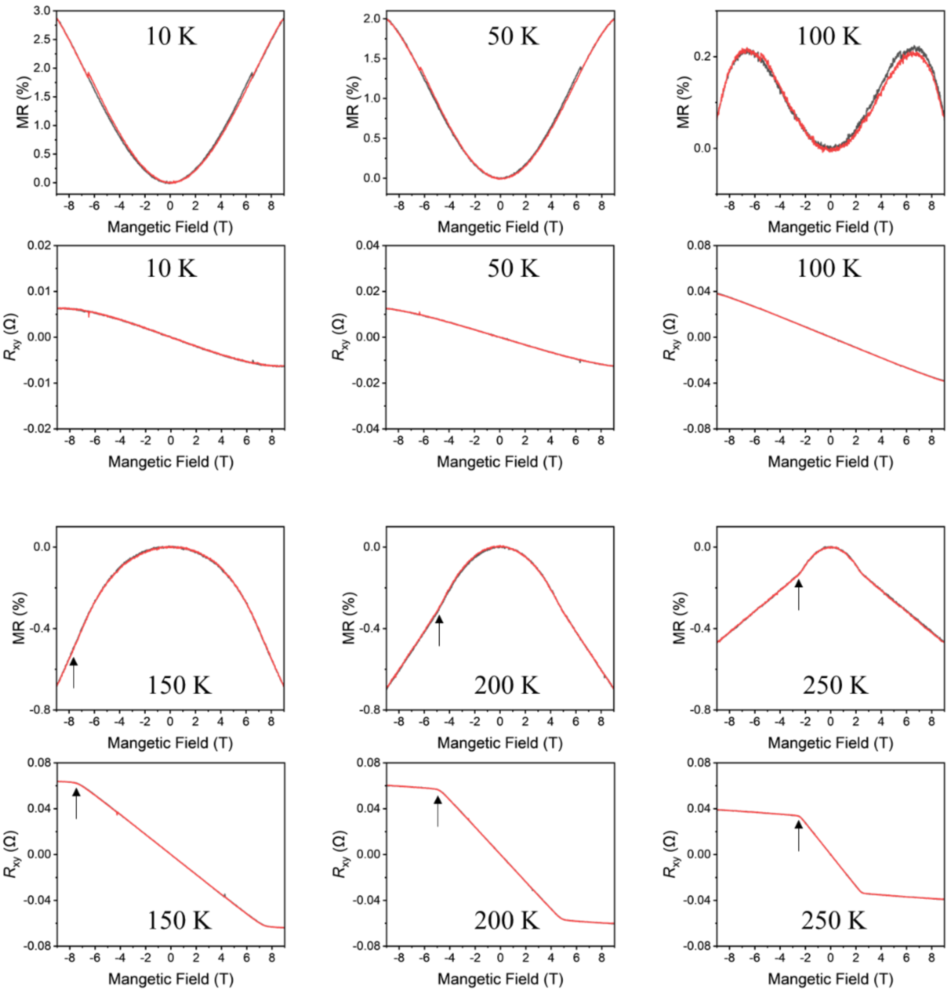

Figure S22. Magneto-resistance (MR) and Hall resistance (R_{xy}) curves of $Fe_{1.5}Se_2$ at different temperatures. Black lines are swept forward from the negative magnetic field to the positive magnetic field, and red lines are swept backward. The arrows indicate the saturation magnetic field.

When the magnetic field is larger than the saturation field, $Fe_{1.5}Se_2$ shows negative MR, because spin-dependent carrier scattering is suppressed. When the magnetic field is smaller than the saturation field, $Fe_{1.5}Se_2$ exhibits the crossover from negative MR to positive MR by decreasing temperatures. This sign change may be ascribed to the transition from magnetism dominated (negative) MR to half-metallic nature dominated (positive) MR.[16] In a half metal, electron-magnon scatterings freeze out exponentially at low temperatures owing to the gapped minority spin states at the Fermi level, generating positive MR behavior.[14,17]There are small resistance jumps in both Hall and MR curves, which may be caused by the magnetic domains flipping from the in-plane direction (easy axis) to the out-of-plane magnetic field direction.

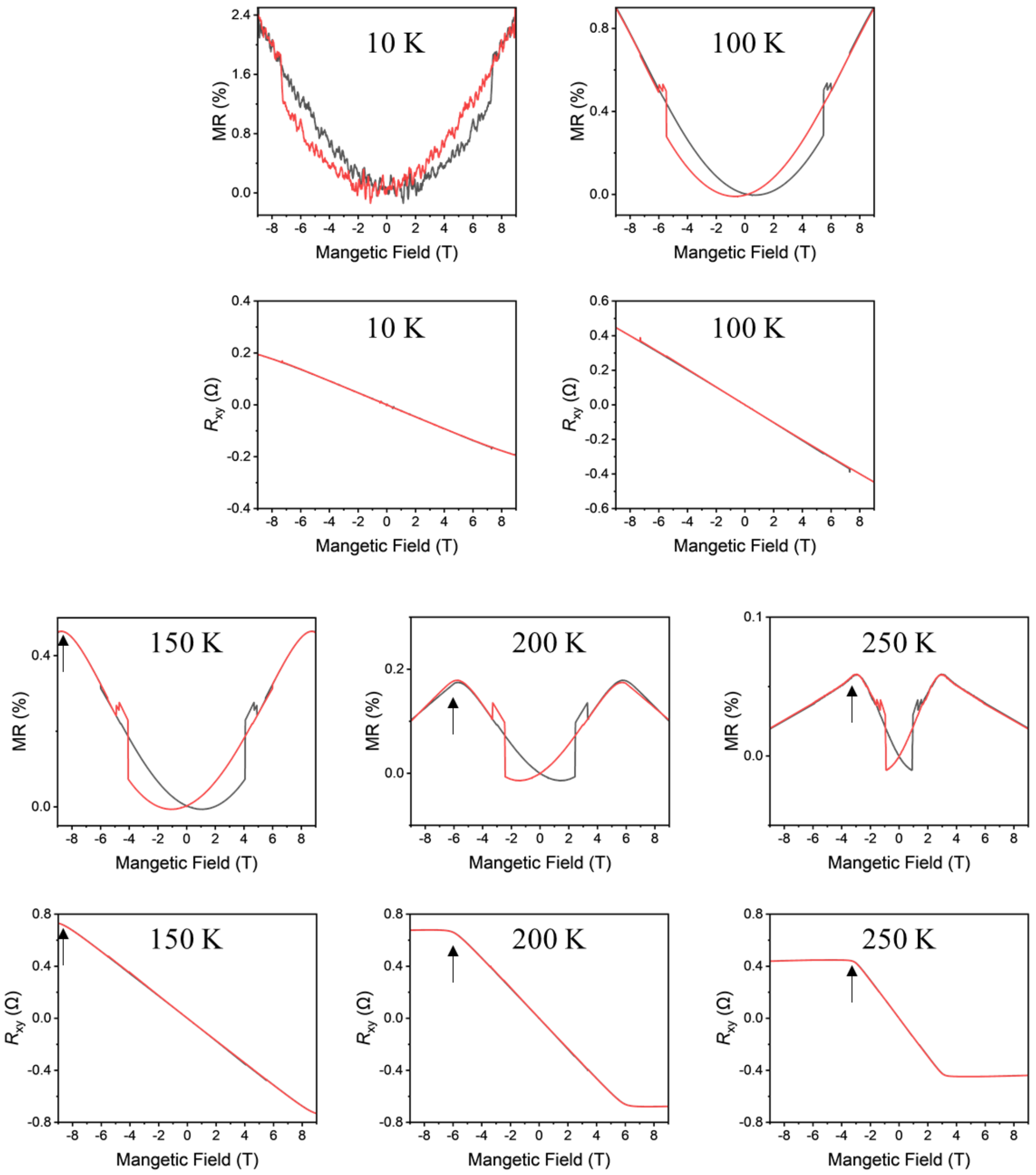

Figure S23. MR and R_{xy} curves of $Fe_{1.6}Se_2$ at different temperatures. Black lines are swept forward from the negative magnetic field to the positive magnetic field, and red lines are swept backward. The arrows indicate the saturation magnetic field.

Compared with $Fe_{1.5}Se_2$, $Fe_{1.6}Se_2$ has more resistance steps than $Fe_{1.5}Se_2$, which may be related to the more complicated magnetic structure of $Fe_{1.6}Se_2$, as indicated by the multi-domain states of its MFM results. Moreover, $Fe_{1.6}Se_2$ shows positive MR below the saturation magnetic field in all temperatures, indicating the increased spin gap, which is in line with the DFT calculations.

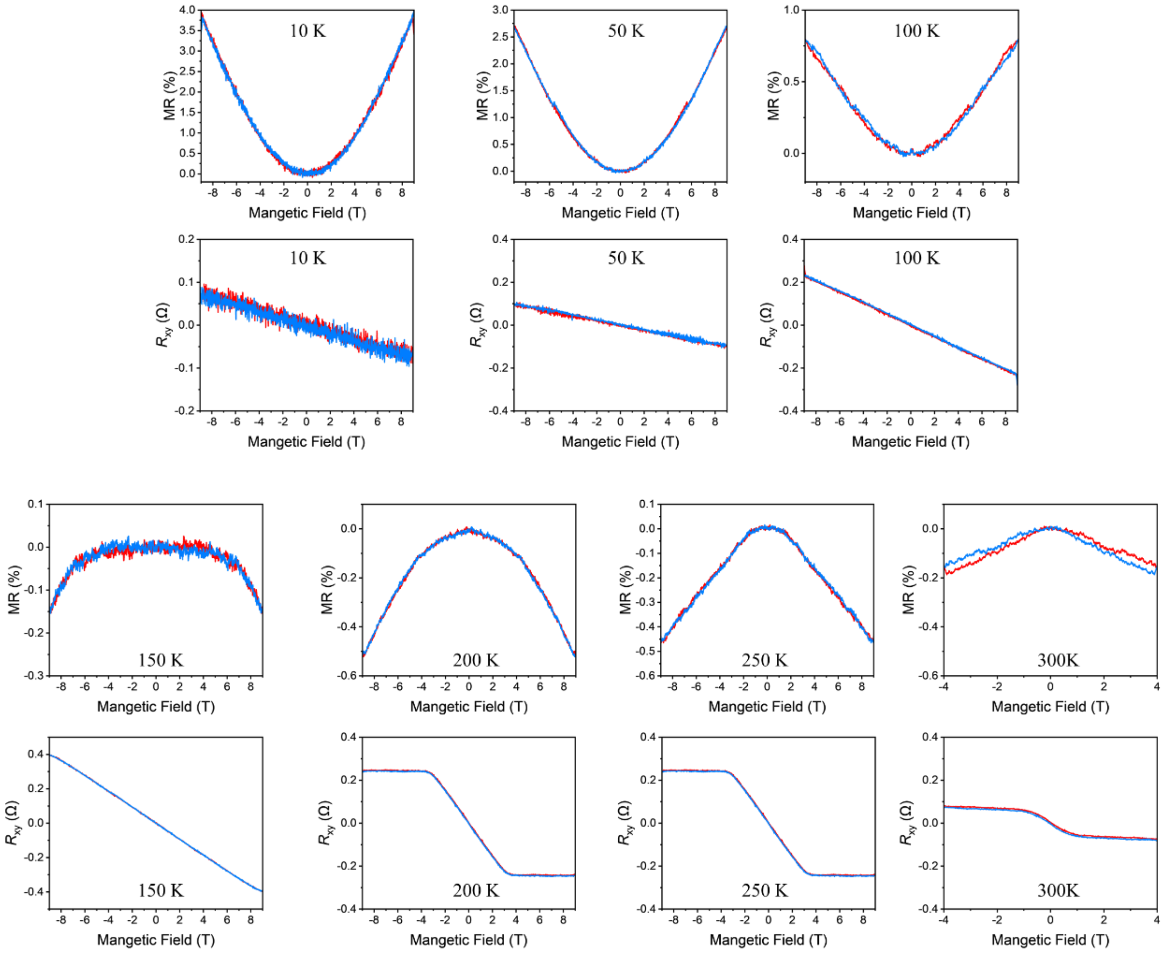

Figure S24. MR and R_{xy} curves of another $Fe_{1.5}Se_2$ nanoflake at different temperatures. Red lines are swept forward from the negative magnetic field to the positive magnetic field, and blue lines are swept backward.

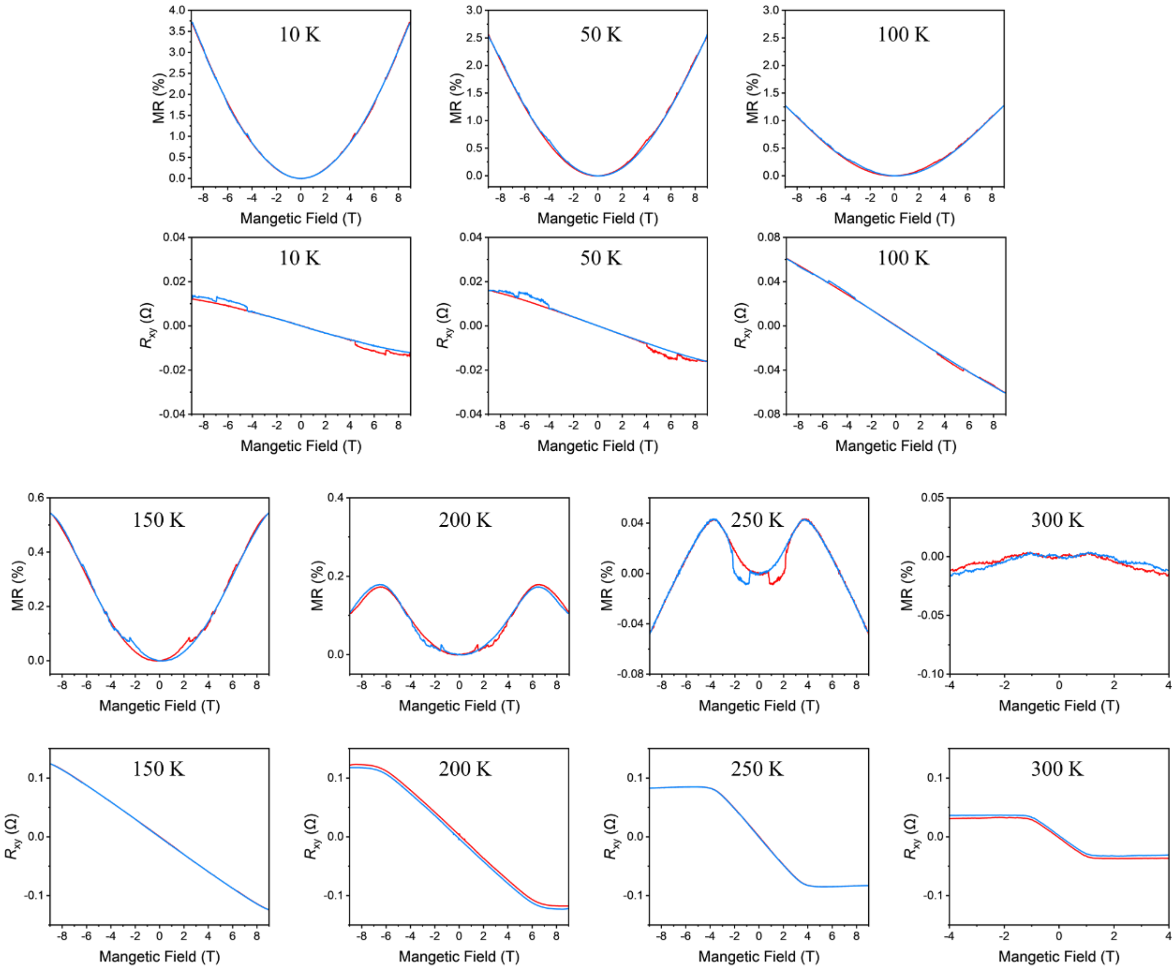

Figure S25. MR and R_{xy} curves of another $Fe_{1.6}Se_2$ nanoflake at different temperatures. Red lines are swept forward from the negative magnetic field to the positive magnetic field, and blue lines are swept backward.

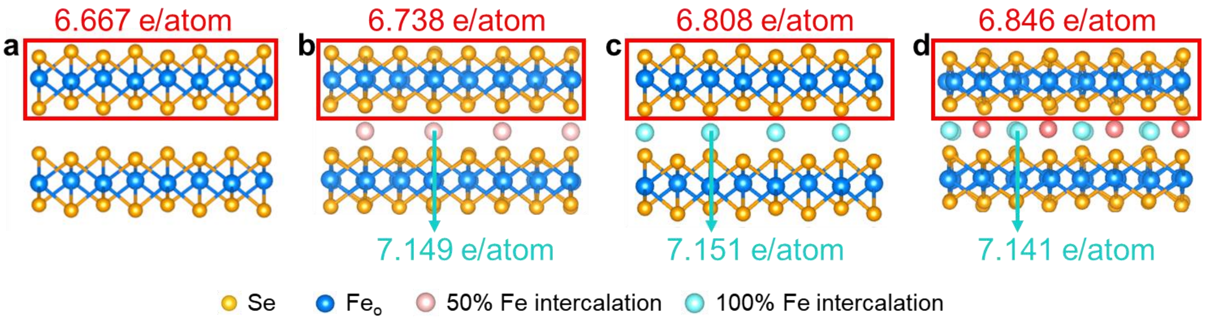

Figure S26. Distribution of averaged Bader charge of $FeSe_2$ backbone (highlighted in red rectangles) and intercalated Fe atoms (highlighted in blue arrows) in $FeSe_2$ (a), $Fe_{1.25}Se_2$ (b), $Fe_{1.5}Se_2$ (c), $Fe_{1.75}Se_2$ (d), respectively.

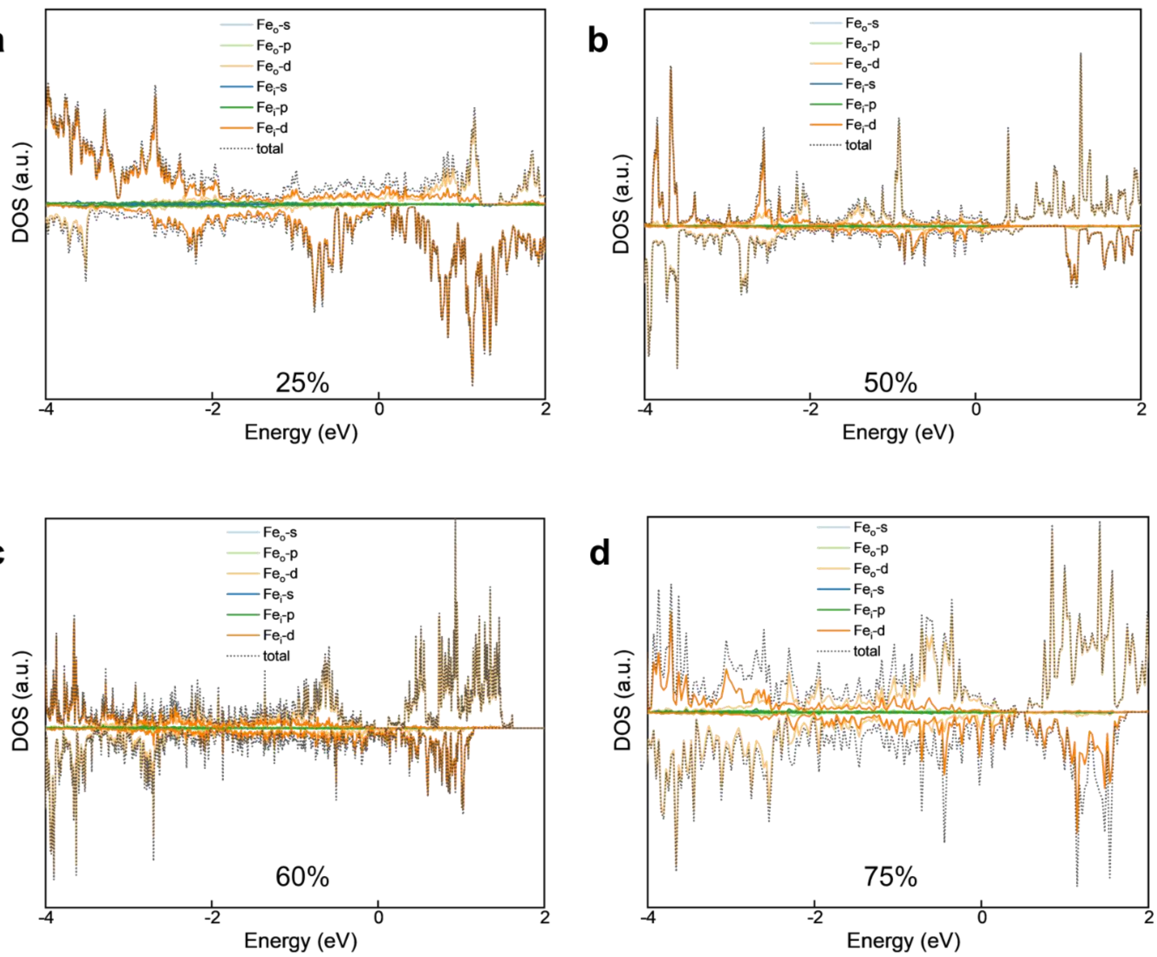

Figure S27. Orbital-resolved DOS of Fe_i and Fe_o in $Fe_{1.25}Se_2$ (a), $Fe_{1.5}Se_2$ (b), $Fe_{1.6}Se_2$ (c), and $Fe_{1.75}Se_2$ (d).

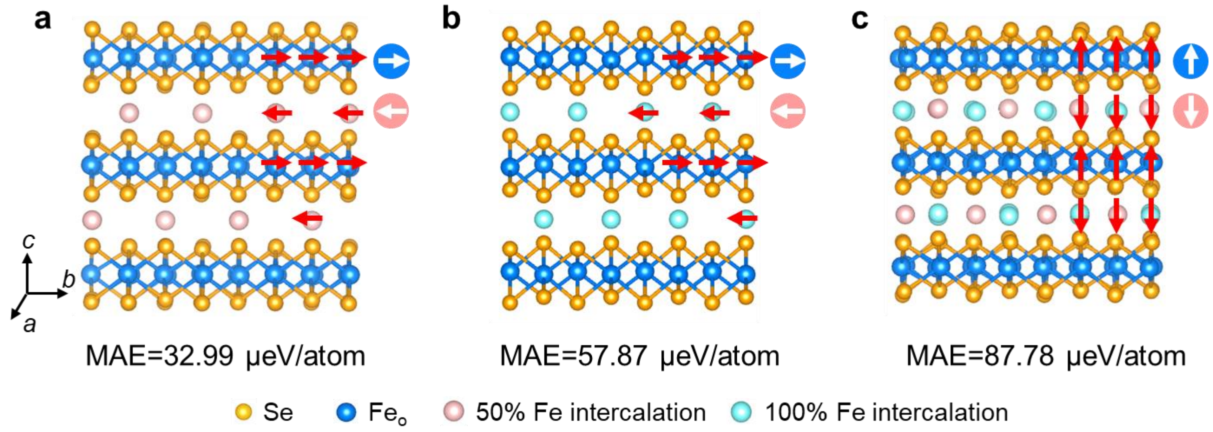

Figure S28. The spin directions of $Fe_{1.25}Se_2$ (a), $Fe_{1.5}Se_2$ (b), and $Fe_{1.75}Se_2$ (c). MAE represents the magnetocrystalline anisotropy energy.

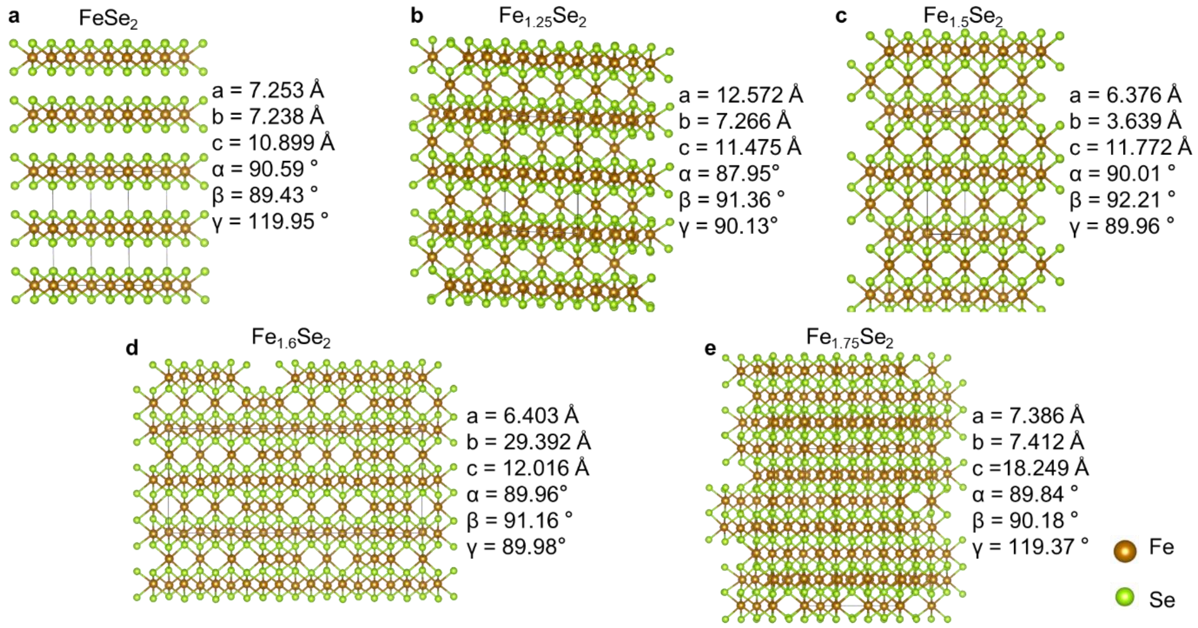

Figure S29. The structure parameters of DFT calculations in $FeSe_2$ (a), $Fe_{1.25}Se_2$ (b), $Fe_{1.5}Se_2$ (c), $Fe_{1.6}Se_2$ (d), $Fe_{1.75}Se_2$ (e), respectively.

Table S1. The comparison of conductivity in 2D materials.

Materials	Conductivity (S m^{-1})
VS_2[18]	~$3.4*10^{5}$
VSe_2[19]	~$1.1*10^{6}$
$NbSe_2$[20]	~$4*10^{5}$
$TaSe_{2-x}S_x$[21]	~$5*10^{5}$
CoSe[22]	~$6*10^{5}$
$Fe_{1.5}Se_2$ and $Fe_{1.6}Se_2$ in this work	~$5*10^{5}$ – $1.0*10^{6}$

Table S2. The calculated magnetic moments of $Fe_{1+x}Se_2$

Materials	The magnetic moment of Fe_o (μ_B per Fe)	The magnetic moment of Fe_i (μ_B per Fe)
$Fe_{1.25}Se_2$	3.19	-3.27
$Fe_{1.5}Se_2$	3.24	-3.37
$Fe_{1.6}Se_2$	3.33	-3.35
$Fe_{1.75}Se_2$	3.36	-3.29

Table S3 The detailed growth conditions of $Fe_{1+x}Se_2$

Materials	The volatilization temperature of Se	The mass of Se	The growth temperature	The mass of $FeCl_2$
$Fe_{1.18}Se_2$	480°C	600 mg	580°C	20 mg
$Fe_{1.25}Se_2$	430°C	600 mg	580°C	20 mg
$Fe_{1.5}Se_2$	400°C	100 mg	580°C	20 mg
$Fe_{1.6}Se_2$	360°C	100 mg	580°C	20 mg
$Fe_{1.75}Se_2$	320°C	50 mg	580°C	20 mg

References

1. Wang S, Rong Y, Fan Y *et al.* Shape Evolution of Monolayer MoS_2 Crystals Grown by Chemical Vapor Deposition. *Chem Mater* 2014; **26**: 6371.
2. Liu HandXue Y Van Der Waals Epitaxial Growth and Phase Transition of Layered $FeSe_2$ Nanocrystals. *Adv Mater* 2021; **33**: 2008456.
3. Long G, Zhang H, Li D *et al.* Magnetic anisotropy and coercivity of Fe_3Se_4 nanostructures. *Appl Phys Lett* 2011; **99**: 202103.
4. Sun X, Zhao S, Bachmatiuk A *et al.* 2D Intrinsic Ferromagnetic MnP Single Crystals. *Small* 2020; **16**: 2001484.
5. Fei Z, Huang B, Malinowski P *et al.* Two-dimensional itinerant ferromagnetism in atomically thin Fe_3GeTe_2. *Nat Mater* 2018; **17**: 778.
6. Zhang X, Lu Q, Liu W *et al.* Room-temperature intrinsic ferromagnetism in epitaxial $CrTe_2$ ultrathin films. *Nat Commun* 2021; **12**: 2492.
7. Wu H, Zhang W, Yang L *et al.* Strong intrinsic room-temperature ferromagnetism in freestanding non-van der Waals ultrathin 2D crystals. *Nat Commun* 2021; **12**: 5688.
8. Bonilla M, Kolekar S, Ma Y *et al.* Strong room-temperature ferromagnetism in VSe_2 monolayers on van der Waals substrates. *Nat Nanotechnol* 2018; **13**: 289.
9. Kang L, Ye C, Zhao X *et al.* Phase-controllable growth of ultrathin 2D magnetic FeTe crystals. *Nat Commun* 2020; **11**: 3729.
10. Kamimura T On the Spin Axis Transition in Fe_7Se_8 (3c). *J Phys Soc Jpn* 1977; **43**: 1594.
11. Jiang J, Cheng R, Feng W *et al.* Van der Waals Epitaxy Growth of 2D Single-Element Room-Temperature Ferromagnet. *Adv Mater* 2023; **35**: 2211701.
12. Wang P, Ge J, Luo J *et al.* Interisland-Distance-Mediated Growth of Centimeter-Scale Two-Dimensional Magnetic Fe_3O_4 Arrays with Unidirectional Domain Orientations. *Nano Lett* 2023; **23**: 1758.
13. Cheng R, Yin L, Wen Y *et al.* Ultrathin ferrite nanosheets for room-temperature two-dimensional magnetic semiconductors. *Nat Commun* 2022; **13**: 5241.
14. Du Y, Xu G Z, Zhang X M *et al.* Crossover of magnetoresistance in the zero-gap half-metallic Heusler alloy Fe_2CoSi. *Europhys Lett* 2013; **103**: 37011.
15. Bombor D, Blum C G F, Volkonskiy O *et al.* Half-Metallic Ferromagnetism with Unexpectedly Small Spin Splitting in the Heusler Compound Co_2FeSi. *Phys Rev Lett* 2013; **110**: 066601.
16. Tewari G C, Srivastava D, Pohjonen R *et al.* Fe_3Se_4: a possible ferrimagnetic half-metal? *J Phys Condens Matter* 2020; **32**: 455801.

17. Ouardi S, Fecher G H, Felser C *et al.* Realization of Spin Gapless Semiconductors: The Heusler Compound Mn_2CoAl. *Phys Rev Lett* 2013; **110**: 100401.
18. Ji Q, Li C, Wang J *et al.* Metallic Vanadium Disulfide Nanosheets as a Platform Material for Multifunctional Electrode Applications. *Nano Lett* 2017; **17**: 4908.
19. Zhang Z, Niu J, Yang P *et al.* Van der Waals Epitaxial Growth of 2D Metallic Vanadium Diselenide Single Crystals and their Extra-High Electrical Conductivity. *Adv Mater* 2017; **29**: 1702359.
20. Huang Y H, Chen R S, Zhang J R *et al.* Electronic transport in $NbSe_2$ two-dimensional nanostructures: semiconducting characteristics and photoconductivity. *Nanoscale* 2015; **7**: 18964.
21. Li L, Deng X, Wang Z *et al.* Superconducting order from disorder in 2H-$TaSe_{2-x}S_x$. *npj Quantum Materials* 2017; **2**: 11.
22. Ma H, Wan Z, Li J *et al.* Phase-Tunable Synthesis of Ultrathin Layered Tetragonal CoSe and Nonlayered Hexagonal CoSe Nanoplates. *Adv Mater* 2019; **31**: 1900901.
